# Inhibitor repurposing reveals ALK, LTK, FGFR, RET and TRK kinases as the targets of AZD1480

**DOI:** 10.18632/oncotarget.22674

**Published:** 2017-11-27

**Authors:** Iva Gudernova, Lukas Balek, Miroslav Varecha, Jana Fialova Kucerova, Michaela Kunova Bosakova, Bohumil Fafilek, Veronika Palusova, Stjepan Uldrijan, Lukas Trantirek, Pavel Krejci

**Affiliations:** ^1^ Department of Biology, Faculty of Medicine, 62500 Brno, Czech Republic; ^2^ International Clinical Research Center, St. Anne’s University Hospital, 65691 Brno, Czech Republic; ^3^ Central European Institute of Technology, Masaryk University, 62500 Brno, Czech Republic

**Keywords:** AZD1480, receptor tyrosine kinase, inhibitor, drug repurposing, in-cell profiling

## Abstract

Many tyrosine kinase inhibitors (TKIs) have failed to reach human use due to insufficient activity in clinical trials. However, the failed TKIs may still benefit patients if their other kinase targets are identified by providing treatment focused on syndromes driven by these kinases. Here, we searched for novel targets of AZD1480, an inhibitor of JAK2 kinase that recently failed phase two cancer clinical trials due to a lack of activity. Twenty seven human receptor tyrosine kinases (RTKs) and 153 of their disease-associated mutants were in-cell profiled for activity in the presence of AZD1480 using a newly developed RTK plasmid library. We demonstrate that AZD1480 inhibits ALK, LTK, FGFR1-3, RET and TRKA-C kinases and uncover a physical basis of this specificity. The RTK activity profiling described here facilitates inhibitor repurposing by enabling rapid and efficient identification of novel TKI targets in cells.

## INTRODUCTION

The deregulation of RTK signaling often leads to disease. More than 70 pathologies are currently associated with alterations in the RTK genes, including developmental disorders, metabolic and degenerative syndromes, and cancer [1, 2]. RTKs represent good therapy targets primarily because they may be easily inhibited by chemical inhibitors of tyrosine kinase activity (TKIs). This inhibition creates a treatment opportunity for conditions caused by aberrant kinase signaling. The past 25 years of intensive research in this field however has only brought a few unequivocal successes, such as in chronic myeloid leukemia, where an addiction of tumor cells to the BCR-ABL tyrosine kinase is counteracted by TKIs, resulting in long-lasting disease remission or a cure [3].

Many developed TKIs have failed to attenuate tumor growth in clinical trials, resulting in only 21 TKIs approved for human use thus far [4]. The current situation is well exemplified by RTKs belonging to the fibroblast growth factor receptor (FGFR) family. FGFR signaling is frequently altered in cancer, as mutations, translocations or amplifications involving the *FGFR1-4* genes associate with bladder, breast, ovarian, lung, oral, endometrial and cervical carcinoma, multiple myeloma, melanoma, glioblastoma, astrocytoma and seminoma [5]. More than 20 different FGFR TKIs have been developed since 1998, when the structures of the first ATP-competitive TKIs based on the pyrimidine core were published [6]. A survey of the public databases reveals that at least 16 FGFR TKIs have been evaluated in cancer clinical trials, but none have yet been approved for human use (ClinicalTrails.gov; [7]). Despite the lack of clinical applications, FGFR TKIs are good drugs, especially the latest generation of compounds, such as AZD4547 or BGJ398, which target FGFRs with nanomolar efficiency and display excellent pharmacological properties [8, 9]. Thus, the failure of TKIs in clinical trials may not lie in the drugs themselves but rather in a poor understanding of the biology of the cancers that they were applied to.

The failed TKIs may be given a second chance by repurposing, an approach in which existing therapeutics are assigned to new targets. The failed TKIs may provide a benefit to patients, particularly in those with developmental or metabolic disorders caused by monogenic RTK deregulation, or in pediatric tumors that are driven by mutations in a small number of protein kinase oncogenes [2, 10-13] (listed in Supplementary Table 1). To facilitate TKI repurposing, we developed a plasmid library consisting of 37 human RTKs and 289 of their mutants, enabling identification of novel TKI targets via in-cell profiling of RTK activity [14]. Here, this plasmid library was used to find novel targets of AZD1480, a TKI originally developed as a JAK2 kinase inhibitor [15]. We demonstrate AZD1480 activity against ALK, LTK, FGFR, RET and TRK kinases and uncover the physical basis of this AZD1480 specificity.

## RESULTS AND DISCUSSION

### Generation of the RTK plasmid library

Full-length human RTK cDNAs were cloned into the pcDNA3.1 plasmid and equipped with the C-terminal V5 epitope for western blot quantification with a single V5 antibody. The 37 cloned RTKs belong to 15 families and represented 67% of the 55 known human RTKs [16, 17] (Table 1). The omitted RTKs were the EPH-family RTKs, MUSK, MER, STYK1 and pseudokinase PTK7. Site-directed mutagenesis was used to prepare a series of disease-associated mutants for each of the cloned RTKs, taking advantage of the information available in the OMIM, PubMed, VarSome and Cosmic [18] databases. Table 1 lists the available RTK mutants; the links to the associated pathologies are given in Supplementary Table 2. Kinase-dead (KD) mutants were also prepared, usually by mutating a distinct lysine residue in the catalytic loop that stabilizes the pentavalent transition state of ATP γ-phosphate [19-22] (Supplementary Table 3). The cloned RTKs were verified by sequencing and validated for expression and catalytic activity as described else where [14].

**Table 1:** List of cloned RTKs and their disease-associated and kinase-dead (KD) variants

Family	Kinase	WT	Mutants	KD
ALK	ALK	+	K1062M G1128A R1192P A1200S F1245C R1275Q	I1250T
	LTK	+	D535N S594A R606Q H608Y R678C R678P P686S D705N R810I S811Y W831C L844I	K544M
AXL	AXL	+	R229C K526N P636H V744M E745K G756E Q764R E809K	K567M
	TYRO3	+	N623K G675R A717T	K538M
DDR	DDR1	+	W385C R607Q F866Y R896Q	K655M
	DDR2	+	C580Y I638F D648Y T654I I726R R752C T765P S768R G774E	K608M
EGFR	EGFR	+	R677H G719C G719S D761Y S768I T790M L858R L861Q	D807N
	ERBB2	+	L755S G776V V842I N857S E914K E971G	K753M
	ERBB3	+	G284R S1046N	-
	ERBB4	+	R544W E563K E836K N855K E872K G936R P1033S D1104Y	K751M
FGFR	FGFR1	+	P252R Y374C R576W K656E V664L W666R	K517M
	FGFR2	+	S252W P253R C342R C342Y S373C Y375C C383R N550K K659E	A649T
	FGFR3	+	R248C S249C G370C S371C Y373C G380R F384L A391E M528I N540K K650E K650M	K508M
	FGFR4	+	P136L G388R N535K V550E E681K	K503M
INSR	IGF1R	+	K998R A1206T M1255I 1278S A1347V	-
	INSR	+	S748L R1068W V1086M R1270C E1285K F1298S G1346E	A1162E
	INSRR	+	R1022W S1050Y D1139Y	K1013M
MET	MET	+	N375S C610Y T1010I M1149T T1191I Y1248C Y1253D	K1110A
	RON	+	R470C R631Q E811K R1018G S1064P E1154K A1193D R1231C R1374C	K1114M
PDGFR	CSF1R	+	L301F L301S I794T M875T Q877K A960T Y969H	K616M
	FLT3	+	V592A L668P F691L D835E D835N D835Y I836M I836V	K644M
	KIT	+	V559G G664R D816H D816V D820Y N822Y Y823D V825A E839K	K623M
	PDGFRA	+	W349C R487L V536E Y555C V561D P577S V626M T674I D842V H845Y D846Y Y849S G853D	K627M
	PDGFRB	+	R561C Y589C R604C E651K L658P N666K D844V D850N R919W	K634A
RET	RET	+	C609Y C618G C618R C620S D631Y C634G C634S G691S G691S/R982C E768N V804M R912P M918T	K758M
ROR	ROR1	+	N513T R567G H589R G590R L746I S776N G827E S889P	-
	ROR2	+	S557L D579N D672N A719T P839L	-
ROS	ROS1	+	F2046Y G2066C R2126W F2138L F2138S R2184I	K1980M
RYK	RYK	+	R510C R563Q	-
TIE	TEK	+	R849W Y897C L898F L914F E990K	K855M
	TIE	+	G533E R713C V765M E842K V1006I	K870M
TRK	TRKA	+	A336E R342Q R508W K538A S550Y R780G	G577R
	TRKB	+	S495C E559G P660L V689M R691C R715Q K814R	Y722C
	TRKC	+	E543D H599Y I695V K746T L760I	K572M
VEGFR	VEGFR1	+	L422P F490S G727C S755P R781Q L930F E943K W1260C	K861M
	VEGFR2	+	C482R D832Y V1041M P1147S S1200Y D1259N G1298S	K868M
	VEGFR3	+	G933R P954S H1035R E1106K S1249F	K879M

A list of wild-type (WT) and mutant RTKs cloned into pcDNA3.1 vector, including the kinase-dead (KD) variants. All RTKs are human full-length variants, equipped with C-terminal V5 epitope. The list of human pathologies associated with the RTK mutants is presented by Supplementary Table 2.

### In-cell profiling of RTK activity by luciferase reporter to ERK MAP kinase

**Figure 1:**
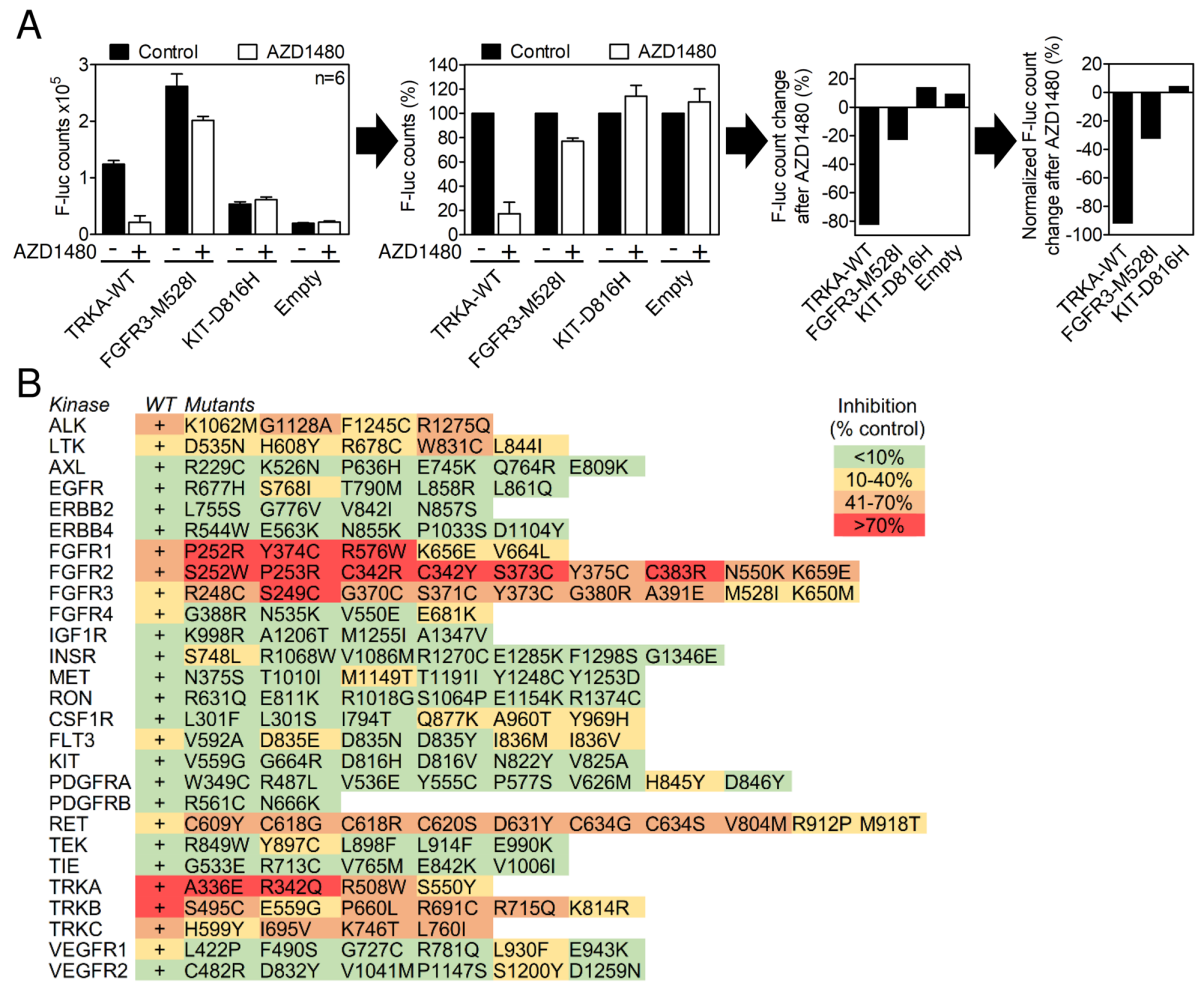
In-cell RTK activity profiling using the SRE-reporter **(A)** 293T cells were transfected with RTK vectors together with the SRE-reporter and treated with 2 μM AZD1480 for 24 hours, and the SRE-reporter transactivation was determined by a firefly luciferase assay. The results are expressed as differences over cells transfected with the SRE-reporter together with an empty plasmid (control). Data are averages from three transfections, each measured twice. **(B)** A total of 27 wild-type (WT) RTKs and 153 of their disease-associated mutants were activity profiled in the presence of AZD1480, as described in (A). Note the AZD1480-mediated inhibition of ALK, LTK, FGFR1-3, RET and TRKA-C; other RTKs were targeted weakly or not targeted.

Most of the RTKs utilize the ERK pathway in their signaling [14]. We therefore evaluated RTK capacity to transactivate the pGL4.33(luc2P/SRE/Hygro) reporter expressing firefly luciferase under the promotor containing serum response elements (SRE-reporter) known to respond to ERK activation [23]. 293T cells were transfected by the RTK vector together with the SRE-reporter and analyzed for RTK-mediated transactivation by luciferase reporter assay 24 hours later (Figure 1A). RTK expression led to their spontaneous activation, except for KIT, FLT3, VEGFR1 and VEGFR2, which required activation by the addition of their cognate ligands, SCF, FLT3 ligand and VEGF. A total of 27 wild-type RTKs and 153 of their disease-associated mutants were capable of SRE-reporter transactivation, which was defined as >3-fold induction of the luciferase signal compared to cells transfected with an empty plasmid (Figure 1B).

### AZD1480 inhibits RTKs belonging to ALK, FGFR, RET and TRK family

AZD1480 was originally developed as a JAK2 kinase inhibitor for treatment of hematological malignancies [15], and it has been reported to inhibit JAK/STAT and FGFR3 signaling in other cell systems [24-26]. It entered clinical trials for solid malignancies in 2010 but was later withdrawn for a lack of clinical activity [27]. The effect of AZD1480 on the activity of SRE-reporter in RTK-expressing 293T cells was determined as a percentage of a luciferase signal compared to cells expressing a given RTK without AZD1480 treatment (Figure 1B). Inhibition less than 10% was considered negligible based on the AZD1480 effect on basal SRE-reporter activity in cells transfected with an empty plasmid instead of RTK (Supplementary Figure 1). AZD1480 significantly inhibited the SRE-reporter transactivation caused by RET, ALK, LTK, FGFR1, FGFR2, FGFR3, TRKA, TRKB and TRKC. Eighteen other RTKs were inhibited weakly or inconsistently (only some variants were inhibited) or were not targeted at all (Figure 1B).

**Figure 2:**
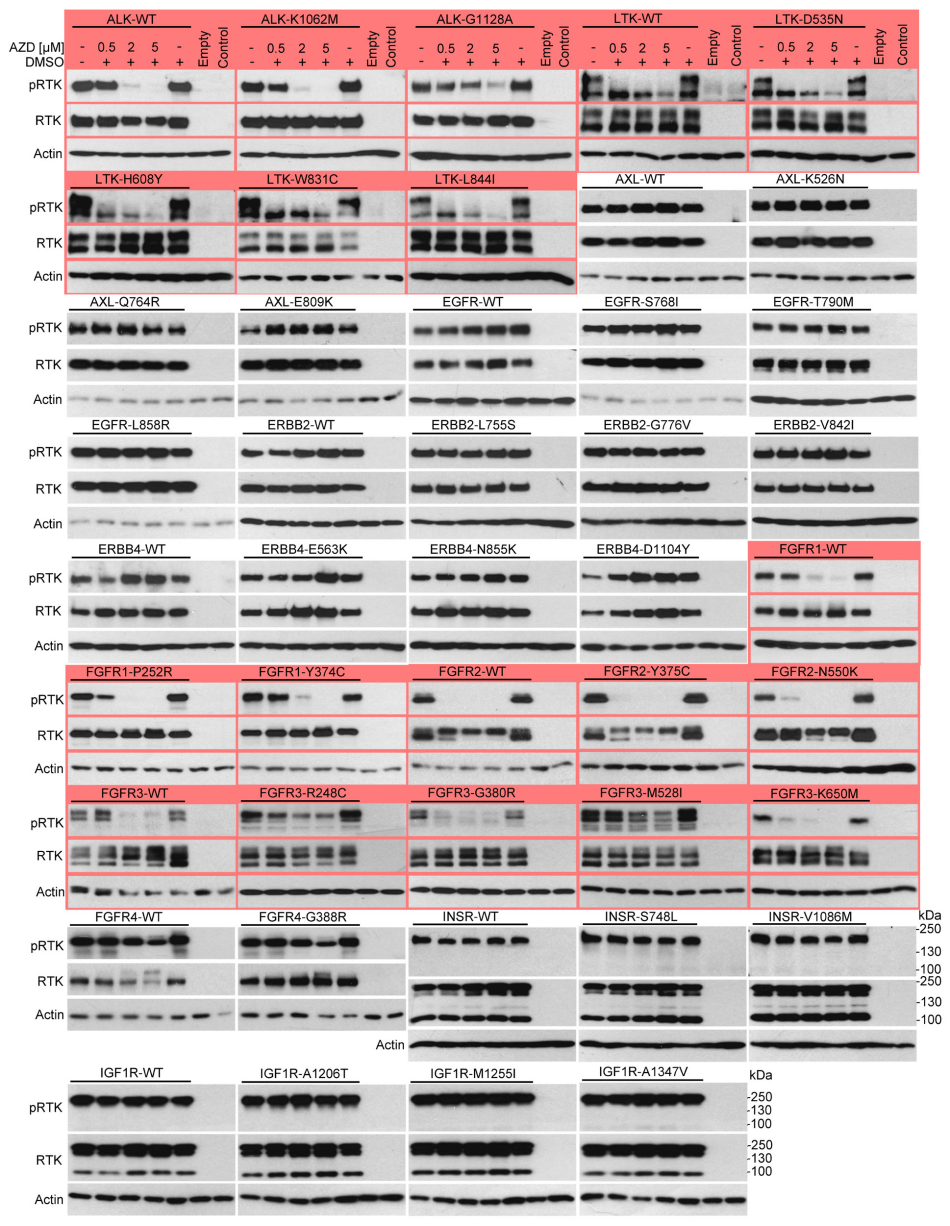
AZD1480 inhibits ALK, LTK and FGFR1-3 activity in cells RTKs were expressed in 293T cells, treated with AZD1480 for 24 hours and analyzed for expression and autophosphorylation (p) by western blot with antibodies recognizing RTK phosphorylation at a specific tyrosine. Note the AZD1480-mediated inhibition of ALK, LTK, FGFR1, FGFR2 and FGFR3 (red). Non-transfected cells (control) or those transfected with an empty plasmid served as controls for transfection, and actin served as a loading control. The expression levels of all RTKs were determined by western blot with the V5 antibody. WT, wild-type.

**Figure 3:**
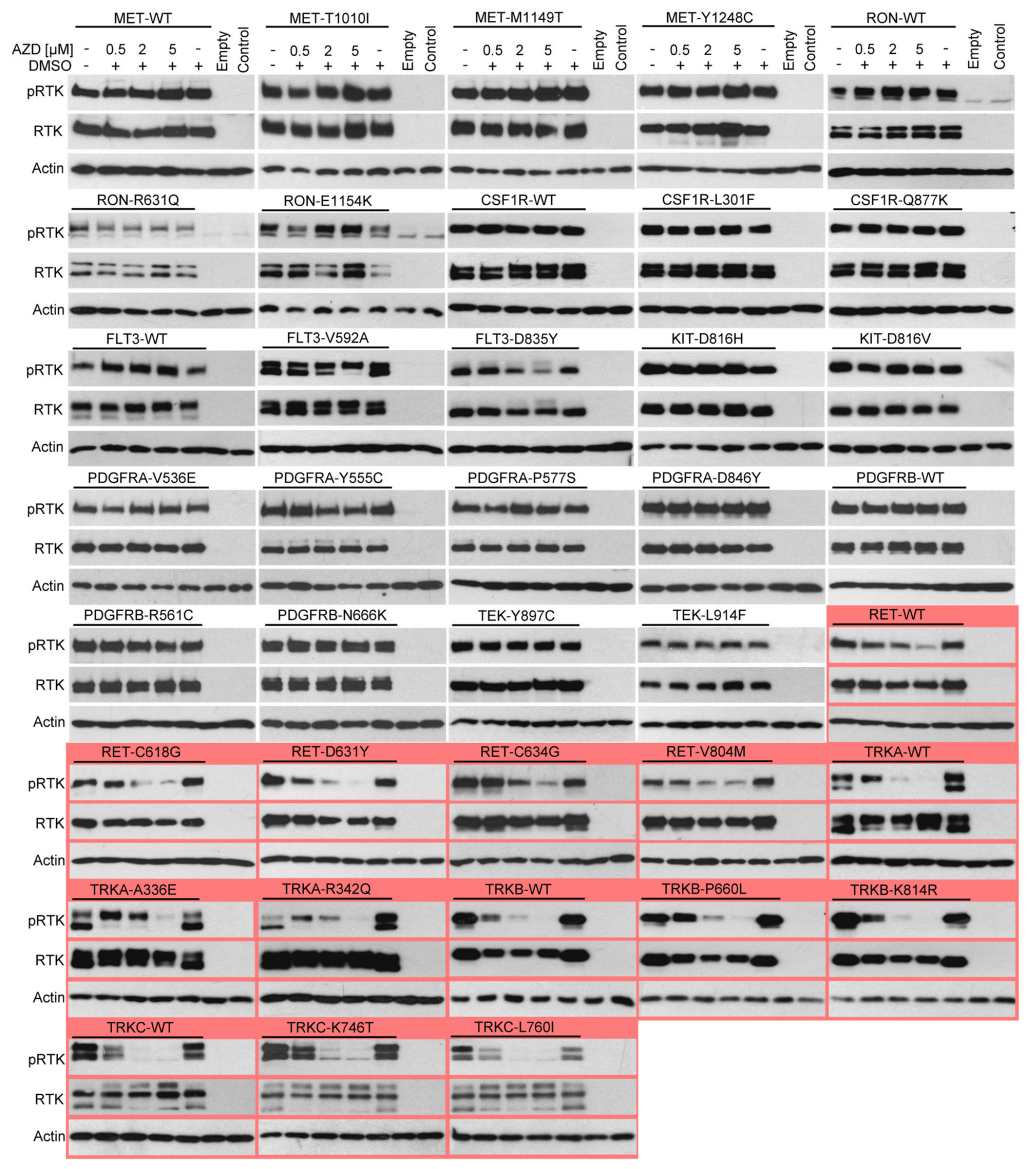
AZD1480 inhibits RET and TRKA-C activity in cells RTKs were expressed in 293T cells, treated with AZD1480 for 24 hours and analyzed for expression and autophosphorylation by western blot with antibodies recognizing RTK phosphorylation at a specific tyrosine. Note the AZD1480-mediated inhibition of RET, TRKA, TRKB and TRKC (red). Non-transfected cells (control) or those transfected with an empty plasmid served as a control for transfection, and actin served as a loading control. The expression levels of all RTKs were determined by western blot with the V5 antibody. WT, wild-type.

Next, a direct determination of the RTK activity was used to confirm the SRE-reporter data. Autophosphorylation at tyrosine residues within the activating loop is a hallmark of spontaneous or ligand-induced RTK activation [28]. 293T cells were transfected with RTK plasmids, exposed to 0.5, 2 and 5 μM AZD1480 for 24 hours and analyzed for RTK phosphorylation by western blot [14]. AZD1480 dosage was based on previously reported inhibition of JAK/STAT signaling in various cell types, which occurred between 0.3-3 μM [15, 29-32]. We considered RTKs in which phosphorylation was either completely suppressed or significantly reduced (>50%) in cells treated with 2 μM AZD1480 to be inhibited. The analysis was carried out with 21 wild-type RTKs and 61 of their mutants (Figures 2, 3). AZD1480 inhibited RET, ALK, LTK, FGFR1, FGFR2, FGFR3, TRKA, TRKB and TRKC; other tested RTKs were not inhibited at all or were only weakly inhibited at 5 μM AZD1480. These findings correspond well to AZD1480 activity in the luciferase assays (Figure 1B), i.e., RTKs inhibited in the SRE-reporter assay were also inhibited in the phosphorylation assays.

### AZD1480 inhibits endogenous ALK, RET, FGFR and TRK signaling

**Figure 4:**
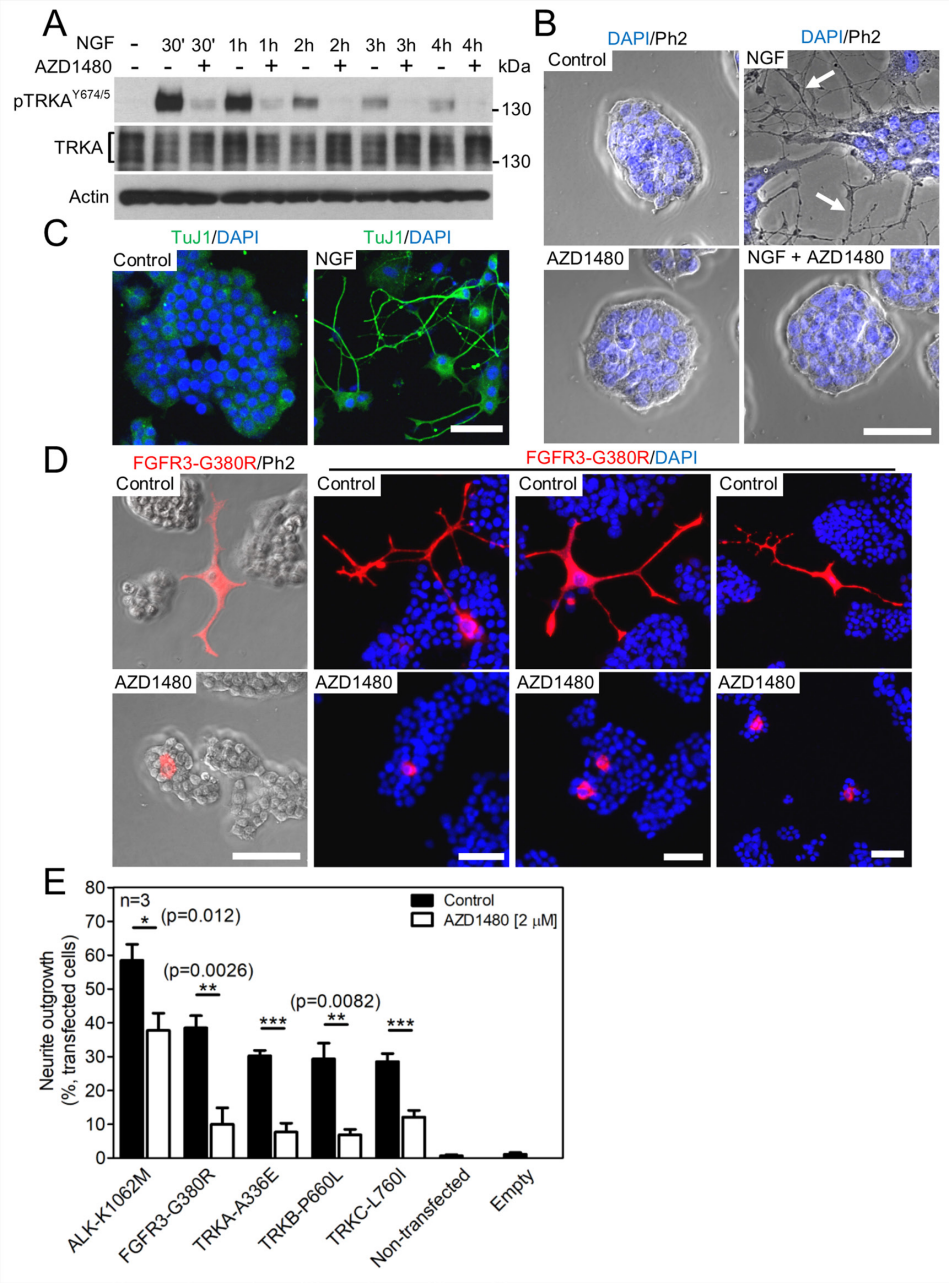
AZD1480 inhibits ALK, FGFR3 and TRKA-C signaling in PC12 cells **(A)** TRKA autophosphorylation (p) in PC12 cells, triggered by treatment with 100 ng/ml of NGF; the NGF effect is rescued by 2 μM AZD1480. Total TRKA and actin levels served as loading controls. **(B)** Neuronal differentiation manifested as neurite outgrowth (arrows) from the colony of PC12 cells, triggered by NGF treatment for seven days; the NGF effect is rescued by AZD1480. **(C)** Immunocytochemistry for a marker of young neurons (TuJ1) in the PC12 cultures treated with NGF for seven days (scale bar of 50 μm). **(D, E)** PC12 cells were transfected with the indicated RTK mutants and grown for seven days, when the RTK-transfected cells were identified by V5 immunocytochemistry and manually scored for neurite outgrowth under a microscope. (D) Examples of neurites in cells transfected with FGFR3-G380R (scale bar of 50 μm). (E) The graph compiles data from three independent experiments (n=3). Approximately 300 cells were examined in each treatment. The data show mean±S.D. (Student’s *t*-test; ^*^p<0.05, ^**^p<0.01, ^***^p<0.001).

The PC12 pheochromocytoma cell line was used as a cell model system to confirm the effect of AZD1480 on RTK activity. When exposed to nerve growth factor (NGF), PC12 cells transition from a proliferative to a differentiated state, which is manifested as the induction of neurite outgrowth driven by TRKA-mediated activation of the ERK MAP kinase pathway [33]. The treatment of cells with NGF triggered TRKA phosphorylation at Y674 and Y675 (Figure 4A), and induced potent neurite outgrowth confirmed by immunocytochemistry for a marker of young neurons, a class III ß-tubulin (TuJ1) [34] (Figure 4B, 4C). Both phenotypes were rescued by AZD1480.

Next, PC12 cells were transfected with ALK, FGFR3, TRKA, TRKB and TRKC mutants associated with neuroblastoma (ALK-K1062M), achondroplasia (FGFR3-G380R), renal carcinoma (TRKA-A336E), early-onset obesity and developmental delay (TRKB-P660L) and colorectal cancer (TRKC-L760I) [35-38]. The transfected cells were visualized by V5 immunocytochemistry detecting the C-terminal V5 tag on the RTKs (Figure 4D). The effect of RTK expression on PC12 differentiation was determined by counting cells with neurites under the microscope, as described in Materials and Methods [39]. Approximately 30-60% of PC12 cells transfected by RTKs produced neurites, and this phenotype was rescued by AZD1480 (Figure 4E).

**Figure 5:**
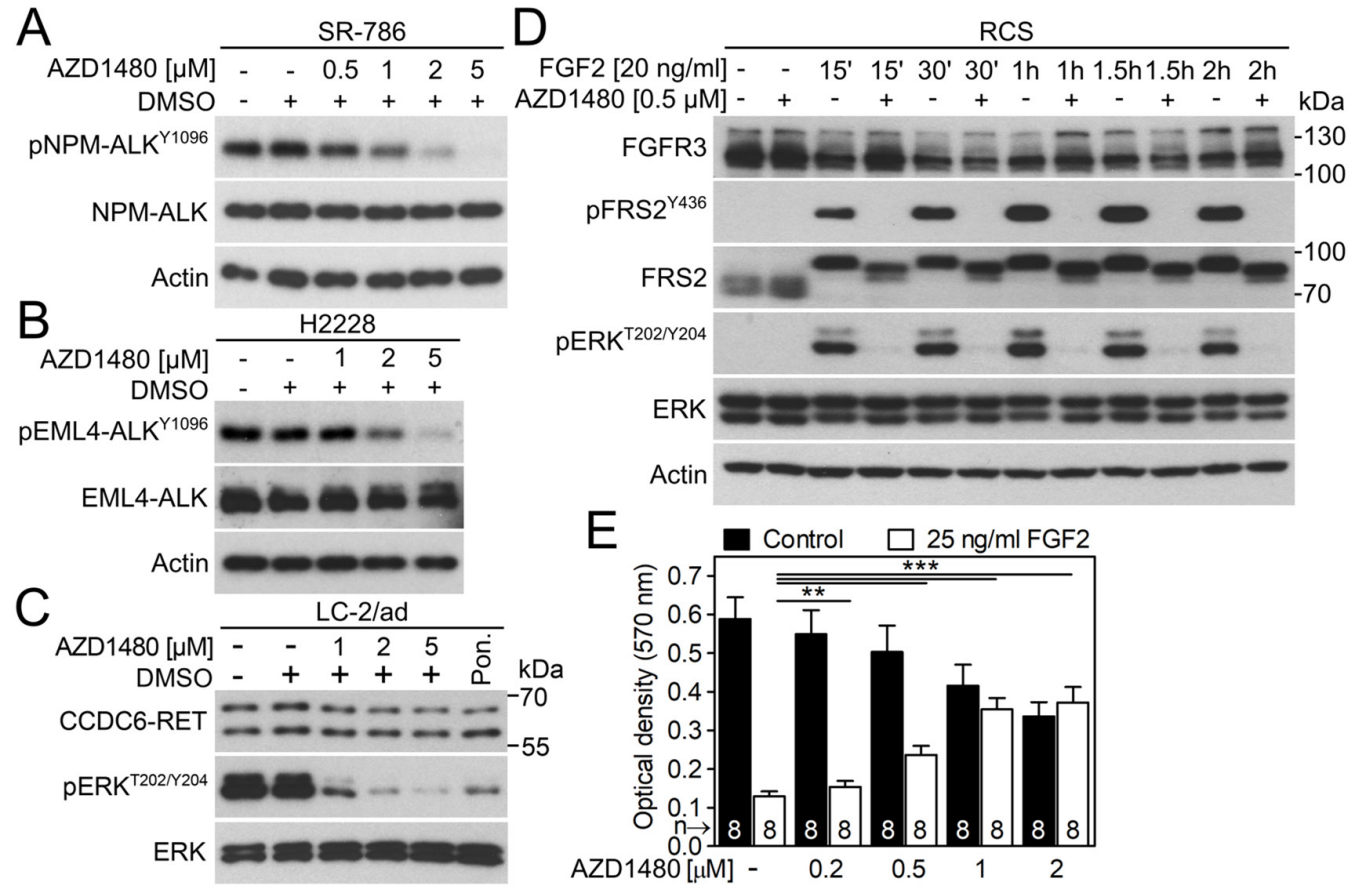
AZD1480 inhibits endogenous ALK, RET and FGFR signaling **(A)** The SR-786 cells expressing NPM-ALK fusion protein were treated with AZD1480 for 12 hours and analyzed for NPM-ALK autophosphorylation (p) by western blot. Total levels of NPM-ALK and actin served as transfection and loading controls. **(B)** H2228 cells expressing EML4-ALK fusion protein were treated with AZD1480 overnight and analyzed for EML4-ALK autophosphorylation (p). **(C)** LC-2/ad cells were treated with AZD1480 for 2 hours and analyzed for phosphorylation (p) of RET target ERK. Total levels of CCDC6-RET and ERK served as loading controls. Ponatinib (0.1 μM; Pon.) was used as a positive control for CCDC6-RET inhibition [52]. **(D)** RCS-F@F cells were treated with AZD1480 for 4 hours before FGF2, and analyzed for FRS2 and ERK phosphorylation (p). Total levels of FGFR3 (detected by a FLAG antibody), FRS2, ERK and actin served as loading controls. **(E)** RCS cells were treated as indicated for 5 days, and the cell numbers were determined by crystal violet staining. Note the profound growth arrest caused by activation of FGFR signaling [53], which is rescued by AZD1480. Data are averages from 8 biological replicates with indicated S.D. (Student’s *t*-test, ^**^p<0.01, ^***^p<0.001).

We used four additional cell models to evaluate the ability of AZD1480 to inhibit endogenous RTK signaling. These cells included SR-786 anaplastic large T cell lymphoma cells expressing the ALK fusion protein NPM-ALK [40], H2228 lung adenocarcinoma cells expressing the ALK fusion protein EML4-ALK [41], LC-2/ad lung adenocarcinoma cells expressing the RET fusion protein CCDC6-RET [42], and RCS chondrosarcoma cells expressing wild-type FGFR2 and FGFR3 [43]. Figure 5A-5C demonstrates AZD1480-mediated inhibition of NPM-ALK and EML4-ALK autophosphorylation and inhibition of ERK activation by CCDC6-RET in LC-2/ad cells. In RCS cells, the activation of FGFR signaling by addition of the FGF ligand (FGF2) resulted in the phosphorylation of ERK and adapter protein FRS2 and triggered potent growth arrest. Both phenotypes were reversed by AZD1480 (Figure 5D, 5E) [44].

### ‘Tight-fit’ binding determines AZD1480 specificity towards RTKs

**Figure 6:**
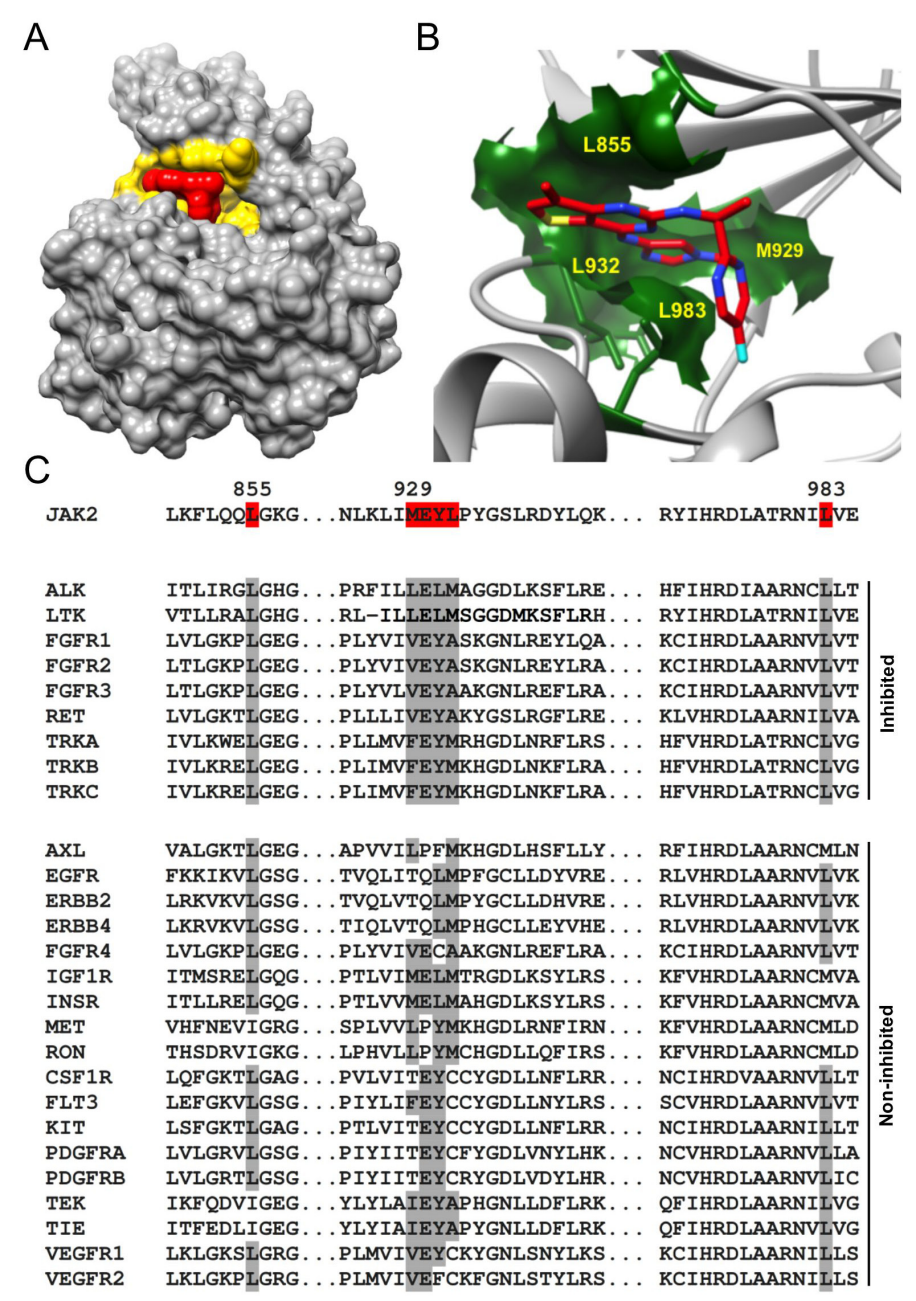
The identity of all amino acids forming the ATP binding pocket determines AZD1480 specificity **(A)** 3D representation of the complex between a catalytic domain of JAK2 and AZD1480 (PDB ID: 2XA4). The JAK2 ATP binding site and AZD1480 are highlighted in yellow and red, respectively. Note the tight AZD1480 fit into its binding site on JAK2, demanding complementarity of shape and charge between JAK2 and AZD1480. **(B)** Magnified view of the AZD1480 binding site in the JAK2-AZD1480 complex (PDB ID: 2XA4). Residues on the outside of the AZD1480 binding site (L855, M929, L932, and L983) are highlighted. **(C)** The sequence alignment of JAK2, RTKs inhibited by AZD1480 and RTKs non inhibited by AZD1480. The residues involved in an interaction between AZD1480 and JAK2 are highlighted in red. Note the differences in the amino acids identities at the position of the AZD1480 binding site (indicated in gray) between inhibited and non-inhibited RTKs.

We next identified the RTK structural/sequence features responsible for AZD1480 specificity. The 3D structure of the complex of JAK2 kinase domain with AZD1480 (PDB ID: 2XA4) [15] shows that AZD1480 binds directly into the ATP binding site of JAK2 (Figure 6A). The obvious tight fit of AZD1480 into its binding site demands high complementarity in both shape and charge between AZD1480 and the six interacting residues (L855, M929-E-Y-L932, and L983) on the surface of JAK2 (Figure 6B). A sequence comparison of JAK2 and RTKs inhibited by AZD1480 with those that were not inhibited revealed that AZD1480’s capacity to inhibit RTKs critically depends on the identity of all amino acids forming the binding pocket (Figure 6C). All inhibited RTKs possessed two Lys residues at positions corresponding to Lys855 and Lys983 in JAK2 and either the LELM, VEYA, or FEYM motif at positions corresponding to the M929-E-Y-L932 motif in the JAK2 sequence. Of note, all non-inhibited RTKs displayed sequence differences at one or more of the positions indicated above.

The AZD1480 activities found here correspond with findings demonstrating AZD1480 activity against FGFR3 and TRKB in experimental models of cancer [26, 45]. Furthermore, our data suggest that some AZD1480 anti-tumor activities that are interpreted as inhibition of JAK/STAT signaling might be mediated by sensitive RTKs instead. For instance, AZD1480 was shown to block the growth of thyroid cancer cells expressing oncogenic RET mutants [46], which may be attributed to the direct inhibition of RET activity.

### Application of the RTK plasmid library for TKI repurposing

Current technologies of RTK profiling are mostly based on cell-free kinase assays utilizing recombinant RTKs with their activity determined by luminescent, fluorescent or radioactive reporters. Cell-free kinase assays are an excellent platform for elucidation of the biochemical properties of TKI-RTK interactions in a binary system containing recombinant kinase and a TKI. These assays are however less suitable for inhibitor profiling against many RTKs.

First, although most available technologies provide good coverage (>40) of the existing wild-type human RTKs, they do not contain many disease-associated mutants [14], which is a serious limitation because an evaluation of inhibitor activity against RTK mutants might identify mutant-specific inhibitors. Preferential inhibition of mutant over wild-type RTK might provide a significant benefit to patients, as demonstrated in lung cancers positive for EGFR-T790M that were treated with inhibitors targeting EGFR-T790M but not wild-type EGFR [47]. Second, activity profiling in cell-free conditions containing only the recombinant kinase, ATP and inhibitor may generate artifacts, such as false positives. This effect is demonstrated by comparing the AZD1480 activity found here with data from cell-free kinase assays utilizing 16 different recombinant RTK kinase domains [44]. Potent AZD1480 activity against IGF1R, INSR and FLT3 was found in cell-free assays, but not in the in-cell profiling carried out here. The false positive inhibitor activities may have occurred because highly active, isolated kinase domains were used in cell-free assays instead of whole RTKs, differences existed in the protein folding between bacterially expressed RTKs versus RTKs expressed in cells, and other factors. These artifacts are difficult to recognize and complicate subsequent inhibitor evaluations. Third, in-cell profiling determines RTK activity in an intact cell environment in the presence of an entire RTK-specific interactome involved in initiation and propagation of the RTK signal and feedback mechanisms regulating this signal. In-cell profiling thus increases the chance to discover inhibitor activities that extend beyond simple targeting of RTK itself. Fourth, cell-free profiling is an initial step in inhibitor evaluation that must be followed by rigorous *in vitro* and *in vivo* testing. From a practical point of view, cell-free RTK profiling may be substituted by in-cell profiling, providing that in-cell technologies offer advantages similar to those of cell-free assays.

Existing in-cell RTK profiling platforms offer good coverage of wild-type RTKs (28-49 human RTKs); however, they do not enable profiling of RTK mutants [14]. The introduction of fast and affordable in-cell RTK profiling technology could eliminate cell-free profiling for inhibitor screening and thus significantly facilitate TKI repurposing. The RTK plasmid library developed here consists of 37 wild-type RTKs and 289 of their mutants associated with more than 70 human pathologies (Table 1, Supplementary Table 1). We demonstrate that when combined with luciferase reporters to record RTK activity, this library enables efficient and rapid identification of novel targets for established TKIs.

## MATERIALS AND METHODS

### Cell culture and crystal violet staining

LC-2/ad (RRID:CVCL_1373) and SR-786 cells (RRID:CVCL_2203) were obtained from DSMZ (Braunschweig, Germany), and H2228 (RRID:CVCL_1543), 293T (RRID:CVCL_0063) and PC12 cells (RRID:CVCL_0481) were obtained from the ATCC (Manassas, VA). RCS-F@F cells expressing endogenous FGFR3 with inserted 3xFLAG epitope were previously described [48]. The cells were propagated in RPMI 1640 (SR-786, H2228), RPMI 1640:F12 (1:1) (LC-2/ad), or DMEM media (293T, PC12, RCS) supplemented with 10% FBS (293T, H2228, LC-2/ad, RCS), 15% FBS (SR-786) or 5% FBS/5% horse serum (PC12) and antibiotics (Invitrogen, Carlsbad, CA). Growth factors and chemicals were obtained from the following manufacturers: SCF, FLT3 ligand, VEGF, NGF, and FGF2 (RnD Systems, Minneapolis, MN); ponatinib and AZD1480 (Selleckchem, Houston, TX). For neuronal differentiation, the PC12 cells were plated on 0.1% gelatin (G1890; Sigma, St. Louis, MO) coated plates at 4x10^5^ cells per well (for 12-well plates). The growth medium was replaced with differentiating medium (DMEM containing 1% horse serum and 100 ng/ml NGF). Cells were cultivated for seven days with fresh media replacement every two days. For crystal violet staining, 200 RCS cells per well were seeded in 96-well plates and treated with AZD1480 and FGF2. After five days, cells were fixed in 4% paraformaldehyde and stained with 0.05% crystal violet in water. Crystal violet was solubilized by 30% acetic acid and quantified by determining the absorbance at 570 nm.

### Plasmid cloning, transfection, neurite outgrowth and luciferase reporter assays

pcDNA3.1 expressing C-terminally V5-tagged RTKs and their mutants were previously described [14]. The pGL4.33(luc2P/SRE/Hygro) vector was from Promega (Madison, WI). 293T cells were transfected using FuGENE6 (Promega). PC12 cells were transfected using Lipofectamine 2000 (Invitrogen) and examined for the presence of neurites at day seven. The number of differentiated cells was determined by visual examination of the cell culture under a microscope. The cells with neurites exceeding at least two cell diameters in length were considered positive [39]. For the luciferase reporter assay, cells were transfected with the RTK plasmid together with the SRE-reporter, and firefly luciferase was determined according to the manufacturer’s protocol (Promega).

### Western blot

Cells were extracted directly to sample buffer (4% SDS, 20% glycerol, 10% β-mercaptoethanol, 0.02% bromophenol blue, 125 mM TRIS-HCl pH 6.8). The samples were resolved by SDS-PAGE, transferred onto a PVDF membrane and visualized by chemiluminescence (Thermo Scientific, Rockford, IL). The following antibodies were used: pFRS2^Y436^ (3861), ALK^Y1096^ (6962), ALK^Y1278/Y1282/Y1283^ (3983), AXL^Y702^ (5724), EGFR^Y992^ (2235), ERBB2^Y877^ (2241), ERBB4^Y984^ (3790), ERK (9102), pERK^T202/Y204^ (4376), FGFR^Y653/Y654^ (3476), FGFR^Y653/Y654^ (3471), IGF1R^Y1135^ (3918), INSR^Y1345^ (3026), MET^Y1234/Y1235^ (3077), MET^Y1003^ (3135), CSF1R^Y699^ (12251), FLT3^Y842^ (4577), KIT^Y703^ (3073), PDGFRA^Y762^ (12022), PDGFRA^Y849^/PDGFRB^Y857^ (3170), RET^Y905^ (3221), TIE2^Y992^ (4221), TRKA^Y674/Y675^/TRKB^Y706/Y707^ (4621), ALK (3333), FGFR4 (2894), RET (3223), and actin (3700) (Cell Signaling, Beverly, MA); VEGFR3^Y1230/Y1231^ (CY1115) (Cell Applications, San Diego, CA); V5 (R960-25) (Invitrogen, Carlsbad, CA); FRS2 (sc-8318), FGFR3 (sc-123) and TRKA (sc-118) (Santa Cruz, Dallas, TX); and FLAG (F1804; Sigma).

### Immunocytochemistry, microscopy and *in silico* modeling

For immunocytochemistry, cells were fixed with 4% paraformaldehyde, exposed to 0.1% Triton X and incubated with TuJ1 (11-264-C100; EXBIO, Vestec, CZ) and V5 (R960-25; Invitrogen) antibody at 4°C overnight. The secondary antibody was Alexa Fluor 568/488 (A11061/A11055; Life Technologies, Grand Island, NY). Images were acquired using confocal inverted microscope Carl Zeiss LSM 700 (Jena, Germany) with 20x air objective as phase contrast combined with the fluorescent signal. The contrast and brightness were adjusted. The JAK2 ATP binding site was identified using the NCBI Conserved Domain Database [49]. A 3D structural representation of the JAK2-AZD1480 complex (PDB ID: 2XA4) was prepared using the UCSF CHIMERA software [50]. T-Coffee [51] was used to generate a sequence alignment of the probed RTKs.

## SUPPLEMENTARY MATERIALS FIGURE AND TABLES








