# Supplementary material for: Inhibitor repurposing reveals ALK, LTK, FGFR, RET and TRK kinases as the targets of AZD1480
Source: Oncotarget. 2017 Nov 27;8(65):109319–31. doi: 10.18632/oncotarget.22674 (PMC5752523; doi:10.18632/oncotarget.22674)
Supplement: Supplementary file 1 [file oncotarget-08-109319-s001.pdf]

Inhibitor repurposing reveals ALK, LTK, FGFR, RET and TRK kinases as the targets of AZD1480

SUPPLEMENTARY MATERIALS

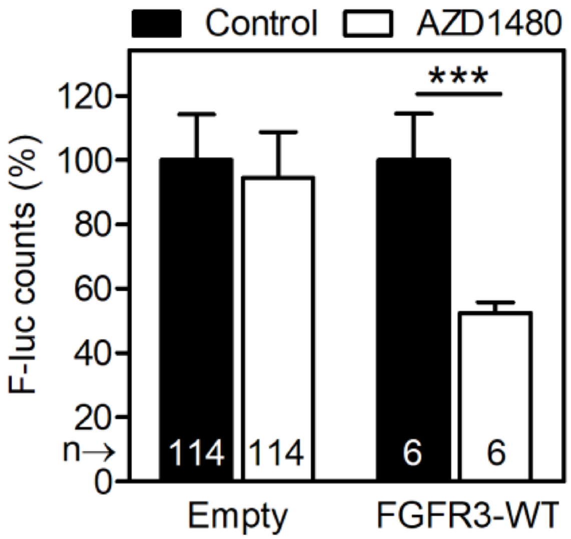

Supplementary Figure 1: AZD1480 effect on basal SRE-reporter activity in 293T cells. 293T cells were transfected with empty pcDNA3.1 vector or with vector expressing wild-type (WT) FGFR3 together with SRE-reporter. Cells were treated with AZD1480 for 24 hours and firefly luciferase signal was determined by luciferase assay. Results are expressed as percentage of luciferase activity in AZD1480-treated cells compared to untreated controls (100%). Data are presented as mean±SD. Student's t-test was used for statistical analysis ($^{***}p<0.001$). n, number of total biological replicates in 19 independent experiments with empty vector and one experiment with wild-type FGFR3.

Supplementary Table 1: Pathologies associated with alterations in RTK genes.

See Supplementary File 1

Supplementary Table 2: Annotated list of mutant RTK variants generated in the study.

See Supplementary File 2

Supplementary Table 3: Kinase-dead RTK mutants generated in the study.

See Supplementary File 3
