# Supplementary material for: Inhibitor repurposing reveals ALK, LTK, FGFR, RET and TRK kinases as the targets of AZD1480
Source: Oncotarget. 2017 Nov 27;8(65):109319–31. doi: 10.18632/oncotarget.22674 (PMC5752523; doi:10.18632/oncotarget.22674)
Supplement: Supplementary file 2 [file oncotarget-08-109319-s002.doc]

Supplementary Table 1: Pathologies associated with alterations in RTK genes

| *RTK* | *Pathology* | *References* |
| --- | --- | --- |
| ALK | Cancer | [1] |
| LTK | Cancer, Systemic lupus erythematosus | [2, 3] |
| AXL | Cancer, Hypogonadotropic hypogonadism | [4, 5] |
| DDR1 | Cancer | [6] |
| DDR2 | Cancer, Spondylometaepiphyseal dysplasia | [7, 8] |
| EGFR | Cancer, Neonatal inflammatory skin and bowel disease | [9, 10] |
| ERBB2 | Cancer | [11] |
| ERBB3 | Cancer, Lethal congenital contractural syndrome | [12, 13] |
| ERBB4 | Cancer, Amyotrophic lateral sclerosis | [14, 15] |
| FGFR1 | Encephalocraniocutaneous lipomatosis, Hartsfield syndrome, Hypogonadotropic hypogonadism, Jackson-Weiss syndrome, Osteoglophonic dysplasia, Pfeiffer syndrome, Trigonocephaly, Kallmann syndrome, Congenital heart disease and sex development disorder | [16-23] |
| FGFR2 | Cancer, Antley-Bixler syndrome, Apert syndrome, Beare-Stevenson cutis gyrate syndrome, Bent bone dysplasia syndrome, Crouzon syndrome, Jackson-Weiss syndrome, LADD syndrome, Pfeiffer syndrome, Saethre-Chotzen syndrome, Scaphocephaly with maxillary retrusion and mental retardation | [24-34] |
| FGFR3 | Cancer, Achondroplasia, CATSHL syndrome, Crouzon syndrome with acanthosis nigricans, Hypochondroplasia, LADD syndrome, Muenke syndrome, Nevus epidermal, SADDAN, Thanatophoric dysplasia type I and II, Seborrheic keratosis | [35-44] |
| IGF1R | Resistance to Insulin-like growth factor I | [45] |
| INSR | Insulin-resistant Diabetes mellitus with acanthosis nigricans, Leprechaunism (Donohue syndrome), Rabson-Mendenhall syndrome | [46-48] |
| MET | Cancer, Deafness | [49, 50] |
| CSF1R | Leukoencephalopathy | [51] |
| FLT3 | Cancer | [52] |
| KIT | Cancer, Mast cell disease, Piebaldism | [53-55] |
| PDGFRA | Cancer | [56] |
| PDGFRB | Basal ganglia calcification, Kosaki overgrowth syndrome, Myofibromatosis, Premature aging syndrome Penttinen type | [57-60] |
| RET | Cancer, Congenital central hypoventilation syndrome, Wagenmann–Froboese syndrome, Renal agenesis, Hirschsprung disease | [61-65] |
| ROR2 | Brachydactyly, Robinow syndrome | [66, 67] |
| RYK | Cleft lip or palate | [68] |
| TEK | Glaucoma, Venous malformations | [69, 70] |
| TRKA | Insensitivity to pain with anhidrosis | [71] |
| TRKB | Obesity with hyperphagia and developmental delay | [72] |
| TRKC | Hirschsprung disease, Congenital heart defects | [73, 74] |
| VEGFR2 | Cancer, Hemangioma capillary infantile | [75, 76] |
| VEGFR3 | Hemangioma capillary infantile, Lymphedema (Milroy disease) | [76, 77] |

References

1. Murugan AK, Xing M. Anaplastic Thyroid cancers harbor novel oncogenic mutations of the ALK gene. Cancer Res. 2011; 71: 4403–11.

2. Kubo T, Kuroda Y, Shimizu H, Kokubu A, Okada N, Hosoda F, Arai Y, Nakamura Y, Taniguchi H, Yanagihara K, Imoto I, Inazawa J, Hirohashi S, et al. Resequencing and copy number analysis of the human tyrosine kinase gene family in poorly differentiated gastric cancer. Carcinogenesis. 2009; 30: 1857–64.

3. Li N, Nakamura K, Jiang Y, Tsurui H, Matsuoka S, Abe M, Ohtsuji M, Nishimura H, Kato K, Kawai T, Atsumi T, Koike T, Shirai T, et al. Gain-of-function polymorphism in mouse and human Ltk: Implications for the pathogenesis of systemic lupus erythematosus. Hum Mol Genet. 2004; 13: 171–9.

4. Chen L, Humphreys A, Turnbull L, Bellini A, Schleiermacher G, Salwen H, Cohn SL, Bown N, Tweddle DA. Identification of different ALK mutations in a pair of neuroblastoma cell lines established at diagnosis and relapse. Oncotarget. 2016; 7: 87301–11.

5. Salian-Mehta S, Xu M, Knox AJ, Plummer L, Slavov D, Taylor M, Bevers S, Hodges RS, Crowley WF, Wierman ME. Functional consequences of AXL sequence variants in hypogonadotropic hypogonadism. J Clin Endocrinol Metab. 2014; 99: 1452–60.

6. Rudd ML, Mohamed H, Price JC, O’Hara AJ, Le Gallo M, Urick ME, Cruz P, Zhang S, Hansen NF, Godwin AK, Sgroi DC, Wolfsberg TG, Mullikin JC, et al. Mutational analysis of the tyrosine kinome in serous and clear cell endometrial cancer uncovers rare somatic mutations in TNK2 and DDR1. BMC Cancer. 2014; 14: 884.

7. Hammerman PS, Sos ML, Ramos AH, Xu C, Dutt A, Zhou W, Brace LE, Woods BA, Lin W, Zhang J, Deng X, Lim SM, Heynck S, et al. Mutations in the DDR2 kinase gene identify a novel therapeutic target in squamous cell lung cancer. Cancer Discov. 2011; 1: 78–89.

8. Bargal R, Cormier-Daire V, Ben-Neriah Z, Le Merrer M, Sosna J, Melki J, Zangen DH, Smithson SF, Borochowitz Z, Belostotsky R, Raas-Rothschild A. Mutations in DDR2 gene cause SMED with short limbs and abnormal calcifications. Am J Hum Genet. 2009; 84: 80–4.

9. Pao W, Miller V, Zakowski M, Doherty J, Politi K, Sarkaria I, Singh B, Heelan R, Rusch V, Fulton L, Mardis E, Kupfer D, Wilson R, et al. EGF receptor gene mutations are common in lung cancers from “never smokers” and are associated with sensitivity of tumors to gefitinib and erlotinib. Proc Natl Acad Sci. 2004; 101: 13306–11.

10. Campbell P, Morton PE, Takeichi T, Salam A, Roberts N, Proudfoot LE, Mellerio JE, Aminu K, Wellington C, Patil SN, Akiyama M, Liu L, McMillan JR, et al. Epithelial inflammation resulting from an inherited loss-of-function mutation in EGFR. J Invest Dermatol. 2014; 134: 2570–8.

11. Stephens P, Hunter C, Bignell G, Edkins S, Davies H, Teague J, Stevens C, O’Meara S, Smith R, Parker A, Barthorpe A, Blow M, Brackenbury L, et al. Lung cancer: intragenic ERBB2 kinase mutations in tumours. Nature. 2004; 431: 525–6.

12. Jaiswal BS, Kljavin NM, Stawiski EW, Chan E, Parikh C, Durinck S, Chaudhuri S, Pujara K, Guillory J, Edgar KA, Janakiraman V, Scholz RP, Bowman KK, et al. Oncogenic ERBB3 mutations in human cancers. Cancer Cell. 2013; 23: 603–17.

13. Narkis G, Ofir R, Manor E, Landau D, Elbedour K, Birk OS. Lethal congenital contractural syndrome type 2 (LCCS2) is caused by a mutation in ERBB3 (Her3), a modulator of the phosphatidylinositol-3-kinase/Akt pathway. Am J Hum Genet. 2007; 81: 589–95.

14. Enkner F, Pichlhöfer B, Zaharie AT, Krunic M, Holper TM, Janik S, Moser B, Schlangen K, Neudert B, Walter K, Migschitz B, Müllauer L. Molecular profiling of thymoma and thymic carcinoma: genetic differences and potential novel therapeutic targets. Pathol Oncol Res. 2016; 23: 551–64.

15. Takahashi Y, Fukuda Y, Yoshimura J, Toyoda A, Kurppa K, Moritoyo H, Belzil V V., Dion PA, Higasa K, Doi K, Ishiura H, Mitsui J, Date H, et al. Erbb4 mutations that disrupt the neuregulin-erbb4 pathway cause amyotrophic lateral sclerosis type 19. Am J Hum Genet. 2013; 93: 900–5.

16. Bennett JT, Tan TY, Alcantara D, Tétrault M, Timms AE, Jensen D, Collins S, Nowaczyk MJM, Lindhurst MJ, Christensen KM, Braddock SR, Brandling-Bennett H, Hennekam RCM, et al. Mosaic activating mutations in FGFR1 cause encephalocraniocutaneous lipomatosis. Am J Hum Genet. 2016; 98: 579–87.

17. Simonis N, Migeotte I, Lambert N, Perazzolo C, de Silva DC, Dimitrov B, Heinrichs C, Janssens S, Kerr B, Mortier G, Van Vliet G, Lepage P, Casimir G, et al. *FGFR1* mutations cause Hartsfield syndrome, the unique association of holoprosencephaly and ectrodactyly. J Med Genet. 2013; 50: 585–92.

18. Dodé C, Levilliers J, Dupont J-M, De Paepe A, Le Dû N, Soussi-Yanicostas N, Coimbra RS, Delmaghani S, Compain-Nouaille S, Baverel F, Pêcheux C, Le Tessier D, Cruaud C, et al. Loss-of-function mutations in FGFR1 cause autosomal dominant Kallmann syndrome. Nat Genet. 2003; 33: 463–5.

19. Roscioli T, Flanagan S, Kumar P, Masel J, Gattas M, Hyland VJ, Glass IA. Clinical findings in a patient with FGFR1 P252R mutation and comparison with the literature. Am J Med Genet. 2000; 93: 22–5.

20. White KE, Cabral JM, Davis SI, Fishburn T, Evans WE, Ichikawa S, Fields J, Yu X, Shaw NJ, McLellan NJ, McKeown C, Fitzpatrick D, Yu K, et al. Mutations that cause osteoglophonic dysplasia define novel roles for FGFR1 in bone elongation. Am J Hum Genet. 2005; 76: 361–7.

21. Muenke M, Schell U, Hehr A, Robin NH, Losken HW, Schinzel A, Pulleyn LJ, Rutland P, Reardon W, Malcolm S, Winter RM. A common mutation in the fibroblast growth factor receptor 1 gene in Pfeiffer syndrome. Nat Genet. 1994; 8: 269–74.

22. Kress W, Petersen B, Collmann H, Grimm T. An unusual FGFR1 mutation ( fibroblast growth factor receptor 1 mutation ) in a girl with non-syndromic trigonocephaly. Cytogenet Cell Genet. 2001; 91: 138–40.

23. Mazen I, Amin H, Kamel A, El Ruby M, Bignon-Topalovic J, Bashamboo A, McElreavey K. Homozygous mutation of the FGFR1 gene associated with congenital heart disease and 46,XY disorder of sex development. Sex Dev. 2016; 10: 16–22.

24. Pollock PM, Gartside MG, Dejeza LC, Powell MA, Mallon MA, Davies H, Mohammadi M, Futreal PA, Stratton MR, Trent JM, Goodfellow PJ. Frequent activating FGFR2 mutations in endometrial carcinomas parallel germline mutations associated with craniosynostosis and skeletal dysplasia syndromes. Oncogene. 2007; 26: 7158–62.

25. Reardon W. Evidence for digenic inheritance in some cases of Antley-Bixler syndrome? J Med Genet. 2000; 37: 26–32.

26. Wilkie AO, Slaney SF, Oldridge M, Poole MD, Ashworth GJ, Hockley AD, Hayward RD, David DJ, Pulleyn LJ, Rutland P. Apert syndrome results from localized mutations of FGFR2 and is allelic with Crouzon syndrome. Nat Genet. 1995; 9: 165–72.

27. Przylepa K a, Paznekas W, Zhang M, Golabi M, Bias W, Bamshad MJ, Carey JC, Hall BD, Stevenson R, Orlow S, Cohen MM, Jabs EW. Fibroblast growth factor receptor 2 mutations in Beare-Stevenson cutis gyrata syndrome. Nat Genet. 1996; 13: 492–4.

28. Merrill AE, Sarukhanov A, Krejci P, Idoni B, Camacho N, Estrada KD, Lyons KM, Deixler H, Robinson H, Chitayat D, Curry CJ, Lachman RS, Wilcox WR, et al. Bent bone dysplasia-FGFR2 type, a distinct skeletal disorder, has deficient canonical FGF signaling. Am J Hum Genet. 2012; 90: 550–7.

29. Reardon W, Winter RM, Rutland P, Pulleyn LJ, Jones BM, Malcolm S. Mutations in the fibroblast growth factor receptor 2 gene cause Crouzon syndrome. Nat Genet. 1994; 8: 98–103.

30. Tartaglia M, Di Rocco C, Lajeunie E, Valeri S, Velardi F, Battaglia PA. Jackson-Weiss syndrome: Identification of two novel FGFR2 missense mutations shared with Crouzon and Pfeiffer craniosynostotic disorders. Hum Genet. 1997; 101: 47–50.

31. Rohmann E, Brunner HG, Kayserili H, Uyguner O, Nürnberg G, Lew ED, Dobbie A, Eswarakumar VP, Uzumcu A, Ulubil-Emeroglu M, Leroy JG, Li Y, Becker C, et al. Mutations in different components of FGF signaling in LADD syndrome. Nat Genet. 2006; 38: 414–7.

32. Rutland P, Pulleyn LJ, Reardon W, Baraitser M, Hayward R, Jones B, Malcolm S, Winter RM, Oldridge M, Slaney SF. Identical mutations in the FGFR2 gene cause both Pfeiffer and Crouzon syndrome phenotypes. Nat Genet. 1995; 9: 173–6.

33. Paznekas W a, Cunningham ML, Howard TD, Korf BR, Lipson MH, Grix a W, Feingold M, Goldberg R, Borochowitz Z, Aleck K, Mulliken J, Yin M, Jabs EW. Genetic heterogeneity of Saethre-Chotzen syndrome, due to TWIST and FGFR mutations. Am J Hum Genet. 1998; 62: 1370–80.

34. McGillivray G, Savarirayan R, Cox TC, Stojkoski C, McNeil R, Bankier A, Bateman JF, Roscioli T, Gardner RJM, Lamande SR. Familial scaphocephaly syndrome caused by a novel mutation in the FGFR2 tyrosine kinase domain. J Med Genet.2005; 42: 656–62.

35. Cappellen D, De Oliveira C, Ricol D, de Medina S, Bourdin J, Sastre-Garau X, Chopin D, Thiery JP, Radvanyi F. Frequent activating mutations of FGFR3 in human bladder and cervix carcinomas. Nat Genet. 1999; 23: 18–20.

36. Shiang R, Thompson LM, Zhu Y-Z, Church DM, Fielder TJ, Bocian M, Winokur ST, Wasmuth JJ. Mutations in the transmembrane domain of FGFR3 cause the most common genetic form of dwarfism, achondroplasia. Cell. 1994; 78: 335–42.

37. Toydemir RM, Brassington AE, Bayrak-Toydemir P, Krakowiak PA, Jorde LB, Whitby FG, Longo N, Viskochil DH, Carey JC, Bamshad MJ. A novel mutation in FGFR3 causes camptodactyly, tall stature, and hearing loss (CATSHL) syndrome. Am J Hum Genet. 2006; 79: 935–41.

38. Meyers GA, Orlow SJ, Munro IR, Przylepa KA, Jabs EW. Fibroblast growth factor receptor 3 (FGFR3) transmembrane mutation in Crouzon syndrome with acanthosis nigricans. Nat Genet.1995; 11: 462–4.

39. Bellus G a, McIntosh I, Smith E a, Aylsworth a S, Kaitila I, Horton W a, Greenhaw G a, Hecht JT, Francomano C a. A recurrent mutation in the tyrosine kinase domain of fibroblast growth factor receptor 3 causes hypochondroplasia. Nat Genet. 1995; 10: 357–9.

40. Bellus GA, Gaudenz K, Zackai EH, Clarke LA, Szabo J, Francomano CA, Muenke M. Identical mutations in three different fibroblast growth factor receptor genes in autosomal dominant craniosynostosis syndromes. Nat Genet. 1996; 14: 174–6.

41. Hafner C, Van Oers JMM, Vogt T, Landthaler M, Stoehr R, Blaszyk H, Hofstaedter F, Zwarthoff EC, Hartmann A. Mosaicism of activating FGFR3 mutations in human skin causes epidermal nevi. J Clin Invest. 2006; 116: 2201–7.

42. Tavormina PL, Bellus GA, Webster MK, Bamshad MJ, Fraley AE, McIntosh I, Szabo J, Jiang W, Jabs EW, Wilcox WR, Wasmuth JJ, Donoghue DJ, Thompson LM, et al. A novel skeletal dysplasia with developmental delay and acanthosis nigricans is caused by a Lys650Met mutation in the fibroblast growth factor receptor 3 gene. Am J Hum Genet. 1999; 64: 722–31.

43. Tavormina PL, Shiang R, Thompson LM, Zhu YZ, Wilkin DJ, Lachman RS, Wilcox WR, Rimoin DL, Cohn DH, Wasmuth JJ. Thanatophoric dysplasia (types I and II) caused by distinct mutations in fibroblast growth factor receptor 3. Nat Genet. 1995; 9: 321–8.

44. Logié A, Dunois-Lardé C, Rosty C, Levrel O, Blanche M, Ribeiro A, Gasc J-M, Jorcano J, Werner S, Satre-Garau X, Thiery JP, Radvanyi F. Activating mutations of the tyrosine kinase receptor FGFR3 are associated with benign skin tumors in mice and humans. Hum Mol Genet. 2005; 14: 1153-60.

45. Abuzzahab MJ, Schneider A, Goddard A, Grigorescu F, Lautier C, Keller E, Kiess W, Klammt J, Kratzsch J, Osgood D, Pfäffle R, Raile K, Seidel B, et al. IGF-I receptor mutations resulting in intrauterine and postnatal growth retardation. N Engl J Med. 2003; 349: 2211–22.

46. Odawara M, Kadowaki T, Yamamoto R, Shibasaki Y, Tobe K, Accili D, Bevins C, Mikami Y, Matsuura N, Akanuma Y. Human diabetes associated with a mutation in the tyrosine kinase domain of the insulin receptor. Science. 1989; 245: 66–8.

47. Kadowaki T, Bevins CL, Cama A, Ojamaa K, Marcus-Samuels B, Kadowaki H, Beitz L, McKeon C, Taylor SI. Two mutant alleles of the insulin receptor gene in a patient with extreme insulin resistance. Science. 1988; 240: 787–90.

48. Kadowaki T, Kadowaki H, Rechler MM, Serrano-Rios M, Roth J, Gorden P, Taylor SI. Five mutant alleles of the insulin receptor gene in patients with genetic forms of insulin resistance. J Clin Invest. 1990; 86: 254–64.

49. Park WS, Dong SM, Kim SY, Na EY, Shin MS, Pi JH, Kim BJ, Bae JH, Hong YK, Lee KS, Lee SH, Yoo NJ, Jang JJ, et al. Somatic mutations in the kinase domain of the Met/hepatocyte growth factor receptor gene in childhood hepatocellular carcinomas. Cancer Res. 1999; 59: 307–10.

50. Mujtaba G, Schultz JM, Imtiaz A, Morell RJ, Friedman TB, Naz S. A mutation of MET, encoding hepatocyte growth factor receptor, is associated with human DFNB97 hearing loss. J Med Genet. 2015; 52: 548–52.

51. Pridans C, Sauter KA, Baer K, Kissel H, Hume DA. CSF1R mutations in hereditary diffuse leukoencephalopathy with spheroids are loss of function. Sci Rep. 2013; 3: 3013.

52. Armstrong SA, Mabon ME, Silverman LB, Li A, Gribben JG, Fox EA, Sallan SE, Korsmeyer SJ. FLT3 mutations in childhood acute lymphoblastic leukemia. Blood. 2004; 103: 3544–6.

53. Hirota S, Isozaki K, Moriyama Y, Hashimoto K, Nishida T, Ishiguro S, Kawano K, Hanada M, Kurata A, Takeda M, Muhammad Tunio G, Matsuzawa Y, Kanakura Y, et al. Gain-of-function mutations of c-kit in human gastrointestinal stromal tumors. Science. 1998; 279: 577–80.

54. Pignon JM, Giraudier S, Duquesnoy P, Jouault H, Imbert M, Vainchenker W, Vernant JP, Tulliez M. A new c-kit mutation in a case of aggressive mast cell disease. Br J Haematol. 1997; 96: 374–6.

55. Giebel LB, Spritz RA. Mutation of the KIT (mast/stem cell growth factor receptor) protooncogene in human piebaldism. Proc Natl Acad Sci. 1991; 88: 8696–9.

56. Heinrich MC, Corless CL, Duensing A, McGreevey L, Chen C-J, Joseph N, Singer S, Griffith DJ, Haley A, Town A, Demetri GD, Fletcher CDM, Fletcher JA. PDGFRA activating mutations in gastrointestinal stromal tumors. Science. 2003; 299: 708–10.

57. Nicolas G, Pottier C, Maltete D, Coutant S, Rovelet-Lecrux A, Legallic S, Rousseau S, Vaschalde Y, Guyant-Marechal L, Augustin J, Martinaud O, Defebvre L, Krystkowiak P, et al. Mutation of the PDGFRB gene as a cause of idiopathic basal ganglia calcification. Neurology. 2013; 80: 181–7.

58. Takenouchi T, Yamaguchi Y, Tanikawa A, Kosaki R, Okano H, Kosaki K. Novel overgrowth syndrome phenotype due to recurrent de novo PDGFRB mutation. J Pediatr. 2015; 166: 483–6.

59. Cheung YH, Gayden T, Campeau PM, LeDuc CA, Russo D, Nguyen V-H, Guo J, Qi M, Guan Y, Albrecht S, Moroz B, Eldin KW, Lu JT, et al. A recurrent PDGFRB mutation causes familial infantile myofibromatosis. Am J Hum Genet. 2013; 92: 996–1000.

60. Johnston JJ, Sanchez-Contreras MY, Keppler-Noreuil KM, Sapp J, Crenshaw M, Finch NCA, Cormier-Daire V, Rademakers R, Sybert VP, Biesecker LG. A point mutation in PDGFRB causes autosomal-dominant Penttinen syndrome. Am J Hum Genet. 2015; 97: 465–74.

61. Mulligan LM, Kwok JB, Healey CS, Elsdon MJ, Eng C, Gardner E, Love DR, Mole SE, Moore JK, Papi L. Germ-line mutations of the RET proto-oncogene in multiple endocrine neoplasia type 2A. Nature. 1993; 363: 458–60.

62. Kanai M, Numakura C, Sasaki A, Shirahata E, Akaba K, Hashimoto M, Hasegawa H, Shirasawa S, Hayasaka K. Congenital central hypoventilation syndrome: a novel mutation of the RET gene in an isolated case. Tohoku J Exp Med. 2002; 196: 241–6.

63. Jasim S, Ying AK, Waguespack SG, Rich TA, Grubbs EG, Jimenez C, Hu MI, Cote G, Habra MA. Multiple endocrine neoplasia type 2B with a RET proto-oncogene A883F mutation displays a more indolent form of medullary thyroid carcinoma compared with a RET M918T mutation. Thyroid. 2011; 21: 189–92.

64. Sugimoto K, Miyazawa T, Nishi H, Miyazaki K, Enya T, Okada M, Takemura T. Heterozygous p.S811F RET gene mutation associated with renal agenesis, oligomeganephronia and total colonic aganglionosis: a case report. BMC Nephrol. 2016; 17: 146.

65. Luo Y, Ceccherini I, Pasini B, Matera I, Bicocchi MP, Barone V, Bocclardi R, Kääriänen H, Weber D, Devoto M, Romeo G. Close linkage with the RET protooncogene and boundaries of deletion mutations in autosomal dominant hirschsprung disease. Hum Mol Genet. 1993; 2: 1803–8.

66. Oldridge M, Fortuna a M, Maringa M, Propping P, Mansour S, Pollitt C, DeChiara TM, Kimble RB, Valenzuela DM, Yancopoulos GD, Wilkie a O. Dominant mutations in ROR2, encoding an orphan receptor tyrosine kinase, cause brachydactyly type B. Nat Genet. 2000; 24: 275–8.

67. Afzal AR, Rajab A, Fenske CD, Oldridge M, Elanko N, Ternes-Pereira E, Tüysüz B, Murday VA, Patton MA, Wilkie AO, Jeffery S. Recessive Robinow syndrome, allelic to dominant brachydactyly type B, is caused by mutation of ROR2. Nat Genet. 2000; 25: 419–22.

68. Watanabe A, Akita S, Tin NTD, Natsume N, Nakano Y, Niikawa N, Uchiyama T, Yoshiura KI. A mutation in RYK is a genetic factor for nonsyndromic cleft lip and palate. Cleft Palate-Craniofacial J. 2006; 43: 310–6.

69. Souma T, Tompson SW, Thomson BR, Siggs OM, Kizhatil K, Yamaguchi S, Feng L, Limviphuvadh V, Whisenhunt KN, Maurer-Stroh S, Yanovitch TL, Kalaydjieva L, Azmanov DN, et al. Angiopoietin receptor TEK mutations underlie primary congenital glaucoma with variable expressivity. J Clin Invest. 2016; 126: 2575–87.

70. Vikkula M, Boon LM, Carraway KL, Calvert JT, Diamonti AJ, Goumnerov B, Pasyk KA, Marchuk DA, Warman ML, Cantley LC, Mulliken JB, Olsen BR. Vascular dysmorphogenesis caused by an activating mutation in the receptor tyrosine kinase TIE2. Cell. 1996; 87: 1181–90.

71. Indo Y, Tsuruta M, Hayashida Y, Karim MA, Ohta K, Kawano T, Mitsubuchi H, Tonoki H, Awaya Y, Matsuda I. Mutations in the TRKA/NGF receptor gene in patients with congenital insensitivity to pain with anhidrosis. Nat Genet. 1996; 13: 485–8.

72. Yeo GSH, Connie Hung C-C, Rochford J, Keogh J, Gray J, Sivaramakrishnan S, O’Rahilly S, Farooqi IS. A de novo mutation affecting human TrkB associated with severe obesity and developmental delay. Nat Neurosci. 2004; 7: 1187–9.

73. Fernández RM, Sánchez-Mejías A, Mena MD, Ruiz-Ferrer M, López-Alonso M, Antiñolo G, Borrego S. A novel point variant in NTRK3, R645C, suggests a role of this gene in the pathogenesis of Hirschsprung disease. Ann Hum Genet. 2009; 73: 19–25.

74. Werner P, Paluru P, Simpson AM, Latney B, Iyer R, Brodeur GM, Goldmuntz E. Mutations in NTRK3 suggest a novel signaling pathway in human congenital heart disease. Hum Mutat. 2014; 35: 1459–68.

75. Antonescu CR, Yoshida A, Guo T, Chang NE, Zhang L, Agaram NP, Qin LX, Brennan MF, Singer S, Maki RG. KDR activating mutations in human angiosarcomas are sensitive to specific kinase inhibitors. Cancer Res. 2009; 69: 7175–9.

76. Walter JW, North PE, Waner M, Mizeracki A, Blei F, Walker JWT, Reinisch JF, Marchuk DA. Somatic mutation of vascular endothelial growth factor receptors in juvenile hemangioma. Genes Chromosomes Cancer. 2002; 33: 295–303.

77. Karkkainen MJ, Ferrell RE, Lawrence EC, Kimak M a, Levinson KL, McTigue M a, Alitalo K, Finegold DN. Missense mutations interfere with VEGFR-3 signalling in primary lymphoedema. Nat Genet. 2000; 25: 153–9.
