# Supplementary material for: Inhibitor repurposing reveals ALK, LTK, FGFR, RET and TRK kinases as the targets of AZD1480
Source: Oncotarget. 2017 Nov 27;8(65):109319–31. doi: 10.18632/oncotarget.22674 (PMC5752523; doi:10.18632/oncotarget.22674)
Supplement: Supplementary file 3 [file oncotarget-08-109319-s003.docx]

Supplementary Table 2: Annotated list of mutant RTK variants generated in the study

| *Family* | *Kinase* | *Mutants* | | | | | | |
| --- | --- | --- | --- | --- | --- | --- | --- | --- |
| ALK | ALK | K1062M [1] | G1128A [2] | R1192P [2] | *A1200S* | F1245C [2] | R1275Q [1,2] |  |
|  | LTK | D535N [3] | S594A | R606Q [4] | *H608Y* | R678C [5] | R678P | P686S [6] |
|  |  | D705N | R810I [7] | S811Y | W831C [8] | L844I [4] |  |  |
| AXL | AXL | R229C | K526N [9] | P636H [10] | V744M | E745K [9] | G756E | *Q764R* |
|  |  | E809K [11] |  |  |  |  |  |  |
|  | TYRO3 | *N623K* | G675R [10] | *A717T* |  |  |  |  |
| DDR | DDR1 | W385C [12] | R607Q [11] | F866Y [12] | R896Q [13] |  |  |  |
|  | DDR2 | C580Y [14] | I638F [14] | *D648Y* | T654I [14] | I726R [15] | R752C [15] | T765P [14] |
|  |  | S768R [14] | G774E [14] |  |  |  |  |  |
| EGFR | EGFR | R677H [16] | G719C [17] | G719S [18] | D761Y [19] | S768I [20] | T790M [20] | L858R [17] |
|  |  | L861Q [17] |  |  |  |  |  |  |
|  | ERBB2 | L755S [21] | G776V [22] | V842I [23] | N857S [24] | E914K [24] | E971G [25] |  |
|  | ERBB3 | G284R [12] | *S1046N* |  |  |  |  |  |
|  | ERBB4 | R544W [26] | E563K [26] | E836K [26] | *N855K* | E872K [27] | G936R [26] | P1033S [26] |
|  |  | *D1104Y* |  |  |  |  |  |  |
| FGFR | FGFR1 | P252R [28] | Y374C [29] | R576W [30] | K656E [31,32] | V664L [33] | W666R [34] |  |
|  | FGFR2 | S252W [35,36] | P253R [35,37] | C342R [38–40] | C342Y [41] | S373C [36] | Y375C [42] | C383R [36] |
|  |  | N550K [36] | K659E [36] |  |  |  |  |  |
|  | FGFR3 | R248C [43–45] | S249C [43,44] | G370C [44] | S371C [43] | Y373C [44] | G380R [46] | F384L [47] |
|  |  | A391E [48] | M528I [49] | N540K [50] | K650E [43,51] | K650M [51] |  |  |
|  | FGFR4 | P136L [52] | G388R [53] | N535K [54] | V550E [54] | E681K [12] |  |  |
| INSR | IGF1R | *K998R* | A1206T [55] | M1255I [12] | ***d1278S*** | A1347V [33] |  |  |
|  | INSR | *S748L* | R1068W | V1086M | R1270C [56] | E1285K | F1298S [8] | G1346E [12] |
|  | INSRR | R1022W [57] | S1050Y | D1139Y [58] |  |  |  |  |
| MET | MET | N375S [59] | C610Y [60] | T1010I [61] | M1149T [62] | T1191I [63] | Y1248C [62] | Y1253D [64] |
|  | RON | R470C | R631Q [22] | E811K | R1018G [65] | *S1064P* | E1154K [66] | A1193D |
|  |  | R1231C | *R1374C* |  |  |  |  |  |
| PDGFR | CSF1R | L301F [67] | L301S [67] | I794T [68] | M875T [68] | Q877K | A960T [7] | Y969H [67] |
|  | FLT3 | V592A [69] | L668P [70] | F691L [71] | D835E [72] | D835N [72] | D835Y [72] | I836M [73] |
|  |  | I836V [74] |  |  |  |  |  |  |
|  | KIT | V559G [75] | G664R [76] | D816H [77] | D816V [78] | D820Y [79] | N822Y [80] | Y823D [81] |
|  |  | V825A [82] | E839K [83] |  |  |  |  |  |
|  | PDGFRA | W349C | R487L [70] | V536E [84] | Y555C [85,86] | V561D [87] | P577S [88] | V626M |
|  |  | T674I (84) | D842V [87] | H845Y [88] | D846Y [90] | Y849S [91] | G853D [88] |  |
|  | PDGFRB | R561C [92] | *Y589C* | R604C [92] | E651K [93] | L658P [94] | N666K [92] | *D844V* |
|  |  | D850N [95] | R919W [11] |  |  |  |  |  |
| RET | RET | C609Y [96] | *C618G* | C618R [97] | C620S [98] | D631Y [99] | C634G [100] | C634S [101] |
|  |  | G691S [102] | G691S/R982C  [103] | *E768N* | V804M [104] | R912P [105] | M918T [106] |  |
| ROR | ROR1 | N513T | *R567G* | *H589R* | G590R [107] | L746I [108] | S776N | G827E |
|  |  | S889P |  |  |  |  |  |  |
|  | ROR2 | S557L | D579N [109] | D672N [107] | A719T [110] | P839L [8] |  |  |
| ROS | ROS1 | F2046Y | G2066C | R2126W [111] | *F2138L* | F2138S | R2184I [58] |  |
| RYK | RYK | ***R510C*** | ***R563Q*** |  |  |  |  |  |
| TIE | TEK | R849W [112] | Y897C [113] | L898F [9] | L914F [114] | E990K [115] |  |  |
|  | TIE | G533E [26] | R713C [116] | V765M [55] | E842K [111] | V1006I [6] |  |  |
| TRK | TRKA | *A336E* | R342Q [110] | R508W [117] | *K538A* | S550Y [70] | R780G [8] |  |
|  | TRKB | S495C [118] | *E559G* | P660L [119] | V689M | R691C [8] | *R715Q* | K814R [56] |
|  | TRKC | E543D [4] | H599Y [120] | I695V [121] | K746T [122] | L760I [121] |  |  |
| VEGFR | VEGFR1 | *L422P* | *F490S* | G727C [123] | *S755P* | R781Q [124] | E943K [60] | L930F [125] |
|  |  | W1260C |  |  |  |  |  |  |
|  | VEGFR2 | C482R [126] | D832Y | V1041M [127] | P1147S [128] | S1200Y | D1259N [129] | G1298S |
|  | VEGFR3 | G933R [130] | P954S [130] | H1035R [131] | E1106K [132] | S1249F [133] |  |  |

Literature references to mutations are given in parentheses. The mutations listed in Cosmic database (cancer.sanger.ac.uk/cosmic) are underlined while mutations available in VarSome database (varsome.com) are in italics. Substitutions in bold were found in the source cDNA sequences used for RTK cloning.

References

1. Chen Y, Takita J, Choi YL, Kato M, Ohira M, Sanada M, Wang L, Soda M, Kikuchi A, Igarashi T, Nakagawara A, Hayashi Y, Mano H, et al. Oncogenic mutations of ALK kinase in neuroblastoma. Nature. 2008; 455: 971–4.

2. Mossé YP, Laudenslager M, Longo L, Cole KA, Wood A, Attiyeh EF, Laquaglia MJ, Sennett R, Lynch JE, Perri P, Laureys G, Speleman F, Kim C, et al. Identification of ALK as a major familial neuroblastoma predisposition gene. Nature. 2008; 455: 930–5.

3. Hucthagowder V, Meyer R, Mullins C, Nagarajan R, DiPersio JF, Vij R, Tomasson MH, Kulkarni S. Resequencing analysis of the candidate tyrosine kinase and RAS pathway gene families in multiple myeloma. Cancer Genet. 2012; 205: 474–8.

4. Kubo T, Kuroda Y, Shimizu H, Kokubu A, Okada N, Hosoda F, Arai Y, Nakamura Y, Taniguchi H, Yanagihara K, Imoto I, Inazawa J, Hirohashi S, et al. Resequencing and copy number analysis of the human tyrosine kinase gene family in poorly differentiated gastric cancer. Carcinogenesis. 2009; 30: 1857–64.

5. Sato Y, Yoshizato T, Shiraishi Y, Maekawa S, Okuno Y, Kamura T, Shimamura T, Sato-Otsubo A, Nagae G, Suzuki H, Nagata Y, Yoshida K, Kon A, et al. Integrated molecular analysis of clear-cell renal cell carcinoma. Nat Genet. 2013; 45: 860–7.

6. Durinck S, Ho C, Wang NJ, Liao W, Jakkula LR, Collisson EA, Pons J, Chan S-W, Lam ET, Chu C, Park K, Hong S, Hur JS, et al. Temporal dissection of tumorigenesis in primary cancers. Cancer Discov. 2011; 1: 137–43.

7. Seshagiri S, Stawiski EW, Durinck S, Modrusan Z, Storm EE, Conboy CB, Chaudhuri S, Guan Y, Janakiraman V, Jaiswal BS, Guillory J, Ha C, Dijkgraaf GJP, et al. Recurrent R-spondin fusions in colon cancer. Nature. 2012; 488: 660–4.

8. Integrated genomic analyses of ovarian carcinoma. Nature. 2011; 474: 609–15.

9. Kan Z, Jaiswal BS, Stinson J, Janakiraman V, Bhatt D, Stern HM, Yue P, Haverty PM, Bourgon R, Zheng J, Moorhead M, Chaudhuri S, Tomsho LP, et al. Diverse somatic mutation patterns and pathway alterations in human cancers. Nature. 2010; 466: 869–73.

10. Zang ZJ, Ong CK, Cutcutache I, Yu W, Zhang SL, Huang D, Ler LD, Dykema K, Gan A, Tao J, Lim S, Liu Y, Futreal PA, et al. Genetic and structural variation in the gastric cancer kinome revealed through targeted deep sequencing. Cancer Res. 2011; 71: 29–39.

11. Giannakis M, Mu XJ, Shukla SA, Qian ZR, Cohen O, Nishihara R, Bahl S, Cao Y, Amin-Mansour A, Yamauchi M, Sukawa Y, Stewart C, Rosenberg M, et al. Genomic Correlates of Immune-Cell Infiltrates in Colorectal Carcinoma. Cell Rep. Cell Press; 2016; 15: 857–65.

12. Ding L, Getz G, Wheeler DA, Mardis ER, McLellan MD, Cibulskis K, Sougnez C, Greulich H, Muzny DM, Morgan MB, Fulton L, Fulton RS, Zhang Q, et al. Somatic mutations affect key pathways in lung adenocarcinoma. Nature. 2008; 455: 1069–75.

13. Grasso CS, Wu Y-M, Robinson DR, Cao X, Dhanasekaran SM, Khan AP, Quist MJ, Jing X, Lonigro RJ, Brenner JC, Asangani IA, Ateeq B, Chun SY, et al. The mutational landscape of lethal castration-resistant prostate cancer. Nature. 2012; 487: 239–43.

14. Hammerman PS, Sos ML, Ramos AH, Xu C, Dutt A, Zhou W, Brace LE, Woods BA, Lin W, Zhang J, Deng X, Lim SM, Heynck S, et al. Mutations in the DDR2 kinase gene identify a novel therapeutic target in squamous cell lung cancer. Cancer Discov. 2011; 1: 78–89.

15. Bargal R, Cormier-Daire V, Ben-Neriah Z, Le Merrer M, Sosna J, Melki J, Zangen DH, Smithson SF, Borochowitz Z, Belostotsky R, Raas-Rothschild A. Mutations in DDR2 Gene Cause SMED with Short Limbs and Abnormal Calcifications. Am J Hum Genet. 2009; 84: 80–4.

16. Franco-Hernandez C, Martinez-Glez V, Alonso ME, De Campos JM, Isla A, Vaquero J, Gutierrez M, Rey JA. Gene dosage and mutational analyses of EGFR in oligodendrogliomas. Int J Oncol. 2007; 30: 209–15.

17. Lynch TJ, Bell DW, Sordella R, Gurubhagavatula S, Okimoto RA, Brannigan BW, Harris PL, Haserlat SM, Supko JG, Haluska FG, Louis DN, Christiani DC, Settleman J, et al. Activating mutations in the epidermal growth factor receptor underlying responsiveness of non-small-cell lung cancer to gefitinib. N Engl J Med. 2004; 350: 2129–39.

18. Paez JG, Jänne PA, Lee JC, Tracy S, Greulich H, Gabriel S, Herman P, Kaye FJ, Lindeman N, Boggon TJ, Naoki K, Sasaki H, Fujii Y, et al. EGFR mutations in lung cancer: correlation with clinical response to gefitinib therapy. Science (80- ). 2004; 304: 1497–500.

19. Balak MN, Gong Y, Riely GJ, Somwar R, Li AR, Zakowski MF, Chiang A, Yang G, Ouerfelli O, Kris MG, Ladanyi M, Miller VA, Pao W. Novel D761Y and common secondary T790M mutations in epidermal growth factor receptor-mutant lung adenocarcinomas with acquired resistance to kinase inhibitors. Clin Cancer Res. 2006; 12: 6494–501.

20. Kosaka T, Yatabe Y, Endoh H, Kuwano H, Takahashi T, Mitsudomi T. Mutations of the epidermal growth factor receptor gene in lung cancer: Biological and clinical implications. Cancer Res. 2004; 64: 8919–23.

21. Lee JW, Soung YH, Seo SH, Kim SY, Park CH, Wang YP, Park K, Nam SW, Park WS, Kim SH, Lee JY, Yoo NJ, Lee SH. Somatic mutations of ERBB2 kinase domain in gastric, colorectal, and breast carcinomas. Clin Cancer Res. 2006; 12: 57–61.

22. Dulak AM, Stojanov P, Peng S, Lawrence MS, Fox C, Stewart C, Bandla S, Imamura Y, Schumacher SE, Shefler E, McKenna A, Carter SL, Cibulskis K, et al. Exome and whole-genome sequencing of esophageal adenocarcinoma identifies recurrent driver events and mutational complexity. Nat Genet. 2013; 45: 478–86.

23. Lee JW, Soung YH, Kim SY, Park WS, Nam SW, Kim SH, Lee JY, Yoo NJ, Lee SH. ERBB2 kinase domain mutation in a gastric cancer metastasis. APMIS. 2005; 113: 683–7.

24. Stephens P, Hunter C, Bignell G, Edkins S, Davies H, Teague J, Stevens C, O’Meara S, Smith R, Parker A, Barthorpe A, Blow M, Brackenbury L, et al. Lung cancer: intragenic ERBB2 kinase mutations in tumours. Nature. 2004; 431: 525–6.

25. Lin W-L, Kuo W-H, Chen F-L, Lee M-Y, Ruan A, Tyan Y-S, Hsu J-D, Chiang H, Han C-P. Identification of the coexisting HER2 gene amplification and novel mutations in the HER2 protein-overexpressed mucinous epithelial ovarian cancer. Ann Surg Oncol. 2011; 18: 2388–94.

26. Prickett TD, Agrawal NS, Wei X, Yates KE, Lin JC, Wunderlich JR, Cronin JC, Cruz P, Rosenberg SA, Samuels Y. Analysis of the tyrosine kinome in melanoma reveals recurrent mutations in ERBB4. Nat Genet. 2009; 41: 1127–32.

27. Soung YH, Lee JW, Kim SY, Wang YP, Jo KH, Moon SW, Park WS, Nam SW, Lee JY, Yoo NJ, Lee SH. Somatic mutations of the ERBB4 kinase domain in human cancers. Int J cancer. 2006; 118: 1426–9.

28. Roscioli T, Flanagan S, Kumar P, Masel J, Gattas M, Hyland VJ, Glass IA. Clinical findings in a patient with FGFR1 P252R mutation and comparison with the literature. Am J Med Genet. 2000; 93: 22–5.

29. Gallo LH, Nelson KN, Meyer AN, Donoghue DJ. Functions of Fibroblast Growth Factor Receptors in cancer defined by novel translocations and mutations. Cytokine Growth Factor Rev. 2015; 26: 425–49.

30. Rand V, Huang J, Stockwell T, Ferriera S, Buzko O, Levy S, Busam D, Li K, Edwards JB, Eberhart C, Murphy KM, Tsiamouri A, Beeson K, et al. Sequence survey of receptor tyrosine kinases reveals mutations in glioblastomas. Proc Natl Acad Sci U S A. 2005; 102: 14344–9.

31. Bennett JT, Tan TY, Alcantara D, Tétrault M, Timms AE, Jensen D, Collins S, Nowaczyk MJM, Lindhurst MJ, Christensen KM, Braddock SR, Brandling-Bennett H, Hennekam RCM, et al. Mosaic Activating Mutations in FGFR1 Cause Encephalocraniocutaneous Lipomatosis. Am J Hum Genet. 2016; 98: 579–87.

32. Pugh TJ, Weeraratne SD, Archer TC, Pomeranz Krummel DA, Auclair D, Bochicchio J, Carneiro MO, Carter SL, Cibulskis K, Erlich RL, Greulich H, Lawrence MS, Lennon NJ, et al. Medulloblastoma exome sequencing uncovers subtype-specific somatic mutations. Nature. 2012; 488: 106–10.

33. Davies H, Hunter C, Smith R, Stephens P, Greenman C, Bignell G, Teague J, Butler A, Edkins S, Stevens C, Parker A, O’Meara S, Avis T, et al. Somatic mutations of the protein kinase gene family in human lung cancer. Cancer Res. 2005; 65: 7591–5.

34. Dodé C, Levilliers J, Dupont J-M, De Paepe A, Le Dû N, Soussi-Yanicostas N, Coimbra RS, Delmaghani S, Compain-Nouaille S, Baverel F, Pêcheux C, Le Tessier D, Cruaud C, et al. Loss-of-function mutations in FGFR1 cause autosomal dominant Kallmann syndrome. Nat Genet. 2003; 33: 463–5.

35. Yu K, Herr AB, Waksman G, Ornitz DM. Loss of fibroblast growth factor receptor 2 ligand-binding specificity in Apert syndrome. Proc Natl Acad Sci USA. 2000; 97: 14536–41.

36. Pollock PM, Gartside MG, Dejeza LC, Powell MA, Mallon MA, Davies H, Mohammadi M, Futreal PA, Stratton MR, Trent JM, Goodfellow PJ. Frequent activating FGFR2 mutations in endometrial carcinomas parallel germline mutations associated with craniosynostosis and skeletal dysplasia syndromes. Oncogene. 2007; 26: 7158–62.

37. Dutt A, Salvesen HB, Chen T-H, Ramos AH, Onofrio RC, Hatton C, Nicoletti R, Winckler W, Grewal R, Hanna M, Wyhs N, Ziaugra L, Richter DJ, et al. Drug-sensitive FGFR2 mutations in endometrial carcinoma. Proc Natl Acad Sci. 2008; 105: 8713–7.

38. Reardon W, Winter RM, Rutland P, Pulleyn LJ, Jones BM, Malcolm S. Mutations in the fibroblast growth factor receptor 2 gene cause Crouzon syndrome. Nat Genet. 1994; 8: 98–103.

39. Rutland P, Pulleyn LJ, Reardon W, Baraitser M, Hayward R, Jones B, Malcolm S, Winter RM, Oldridge M, Slaney SF. Identical mutations in the FGFR2 gene cause both Pfeiffer and Crouzon syndrome phenotypes. Nat Genet.1995; 9: 173–6.

40. Park WJ, Meyers GA, Li X, Theda C, Day D, Orlow SJ, Jones MC, Jabs EW. Novel FGFR2 mutations in Crouzon and Jackson-Weiss syndromes show allelic heterogeneity and phenotypic variability. Hum Mol Genet. 1995; 4: 1229–33.

41. Eswarakumar VP, Horowitz MC, Locklin R, Morriss-Kay GM, Lonai P. A gain-of-function mutation of Fgfr2c demonstrates the roles of this receptor variant in osteogenesis. Proc Natl Acad Sci U S A. 2004; 101: 12555–60.

42. Wang T-J, Huang C-B, Tsai F-J, Wu J-Y, Lai R-B, Hsiao M. Mutation in the FGFR2 gene in a Taiwanese patient with Beare-Stevenson cutis gyrata syndrome. Clin Genet. Denmark; 2002; 61: 218–21.

43. Tavormina PL, Shiang R, Thompson LM, Zhu YZ, Wilkin DJ, Lachman RS, Wilcox WR, Rimoin DL, Cohn DH, Wasmuth JJ. Thanatophoric dysplasia (types I and II) caused by distinct mutations in fibroblast growth factor receptor 3. Nat Genet. 1995; 9: 321–8.

44. Rousseau F, El Ghouzzi V, Delezoide AL, Legeai-Mallet L, Le Merrer M, Munnich A, Bonaventure J. Missense FGFR3 mutations create cysteine residues in thanatophoric dwarfism type I (TD1). Hum Mol Genet. 1996; 5: 509–12.

45. Naski MC, Wang Q, Xu J, Ornitz DM. Graded activation of fibroblast growth factor receptor 3 by mutations causing achondroplasia and thanatophoric dysplasia. Nat Genet. 1996; 13: 233–7.

46. Bellus GA, Hefferon TW, Ortiz de Luna RI, Hecht JT, Horton WA, Machado M, Kaitila I, McIntosh I, Francomano CA. Achondroplasia is defined by recurrent G380R mutations of FGFR3. Am J Hum Genet. 1995; 56: 368–73.

47. Chesi M, Brents LA, Ely SA, Bais C, Robbiani DF, Mesri EA, Kuehl WM, Bergsagel PL. Activated fibroblast growth factor receptor 3 is an oncogene that contributes to tumor progression in multiple myeloma. Blood. 2001; 97: 729–36.

48. Meyers GA, Orlow SJ, Munro IR, Przylepa KA, Jabs EW. Fibroblast growth factor receptor 3 (FGFR3) transmembrane mutation in Crouzon syndrome with acanthosis nigricans. Nat Genet. 1995; 11: 462–4.

49. Kant SG, Cervenkova I, Balek L, Trantirek L, Santen GW, De Vries MC, Van Duyvenvoorde HA, Van Der Wielen MJR, Verkerk AJMH, Uitterlinden AG, Hannema SE, Wit JM, Oostdijk W, et al. A novel variant of FGFR3 causes proportionate short stature. Eur J Endocrinol. 2015; 172: 763–70.

50. Prinos P, Costa T, Sommer A, Kilpatrick MW, Tsipouras P. A common FGFR3 gene mutation in hypochondroplasia. Hum Mol Genet. 1995; 4: 2097–101.

51. Chesi M, Nardini E, Brents LA, Schrock E, Ried T, Kuehl WM, Bergsagel PL. Frequent translocation t(4;14)(p16.3;q32.3) in multiple myeloma is associated with increased expression and activating mutations of fibroblast growth factor receptor 3. Nat Genet. 1997; 16: 260–4.

52. Peláez-García A, Barderas R, Torres S, Hernández-Varas P, Teixidó J, Bonilla F, de Herreros AG, Casal JI. FGFR4 Role in Epithelial-Mesenchymal Transition and Its Therapeutic Value in Colorectal Cancer. PLoS One. 2013; 8.

53. Bange J, Prechtl D, Cheburkin Y, Specht K, Harbeck N, Schmitt M, Knyazeva T, Müller S, Gärtner S, Sures I, Wang H, Imyanitov E, Häring HU, et al. Cancer progression and tumor cell motility are associated with the FGFR4 Arg388 allele. Cancer Res. 2002; 62: 840–7.

54. Taylor VI JG, Cheuk AT, Tsang PS, Chung JY, Song YK, Desai K, Yu Y, Chen QR, Shah K, Youngblood V, Fang J, Su YK, Yeung C, et al. Identification of FGFR4-activating mutations in human rhabdomyosarcomas that promote metastasis in xenotransplanted models. J Clin Invest. 2009; 119: 3395–407.

55. Quesada V, Conde L, Villamor N, Ordonez GR, Jares P, Bassaganyas L, Ramsay AJ, Bea S, Pinyol M, Martinez-Trillos A, Lopez-Guerra M, Colomer D, Navarro A, et al. Exome sequencing identifies recurrent mutations of the splicing factor SF3B1 gene in chronic lymphocytic leukemia. Nat Genet. 2011; 44: 47–52.

56. Mouradov D, Sloggett C, Jorissen RN, Love CG, Li S, Burgess AW, Arango D, Strausberg RL, Buchanan D, Wormald S, O’Connor L, Wilding JL, Bicknell D, et al. Colorectal cancer cell lines are representative models of the main molecular subtypes of primary cancer. Cancer Res. 2014; 74: 3238–47.

57. Zang ZJ, Cutcutache I, Poon SL, Zhang SL, McPherson JR, Tao J, Rajasegaran V, Heng HL, Deng N, Gan A, Lim KH, Ong CK, Huang D, et al. Exome sequencing of gastric adenocarcinoma identifies recurrent somatic mutations in cell adhesion and chromatin remodeling genes. Nat Genet. 2012; 44: 570–4.

58. Abaan OD, Polley EC, Davis SR, Zhu YJ, Bilke S, Walker RL, Pineda M, Gindin Y, Jiang Y, Reinhold WC, Holbeck SL, Simon RM, Doroshow JH, et al. The exomes of the NCI-60 panel: a genomic resource for cancer biology and systems pharmacology. Cancer Res. 2013; 73: 4372–82.

59. Tabone T, Abuhusain HJ, Nowak AK, Erber WN, McDonald KL. Multigene profiling to identify alternative treatment options for glioblastoma: a pilot study. J Clin Pathol. 2014; 67: 550–5.

60. Prickett TD, Agrawal NS, Wei X, Yates KE, Lin JC, Wunderlich JR, Cronin JC, Cruz P, Rosenberg SA, Samuels Y. Analysis of the tyrosine kinome in melanoma reveals recurrent mutations in ERBB4. Nat Genet. 2009; 41: 1127–32.

61. Ma PC, Kijima T, Maulik G, Fox EA, Sattler M, Griffin JD, Johnson BE, Salgia R. c-MET mutational analysis in small cell lung cancer: novel juxtamembrane domain mutations regulating cytoskeletal functions. Cancer Res. 2003; 63: 6272–81.

62. Schmidt L, Duh FM, Chen F, Kishida T, Glenn G, Choyke P, Scherer SW, Zhuang Z, Lubensky I, Dean M, Allikmets R, Chidambaram A, Bergerheim UR, et al. Germline and somatic mutations in the tyrosine kinase domain of the MET proto-oncogene in papillary renal carcinomas. Nat Genet.1997; 16: 68–73.

63. Park WS, Dong SM, Kim SY, Na EY, Shin MS, Pi JH, Kim BJ, Bae JH, Hong YK, Lee KS, Lee SH, Yoo NJ, Jang JJ, et al. Somatic mutations in the kinase domain of the Met/hepatocyte growth factor receptor gene in childhood hepatocellular carcinomas. Cancer Res.1999; 59: 307–10.

64. Di Renzo MF, Olivero M, Martone T, Maffe A, Maggiora P, Stefani AD, Valente G, Giordano S, Cortesina G, Comoglio PM. Somatic mutations of the MET oncogene are selected during metastatic spread of human HNSC carcinomas. Oncogene. 2000; 19: 1547–55.

65. Catenacci DVT, Cervantes G, Yala S, Nelson EA, El-Hashani E, Kanteti R, El Dinali M, Hasina R, Brägelmann J, Seiwert T, Sanicola M, Henderson L, Grushko TA, et al. RON (MST1R) is a novel prognostic marker and therapeutic target for gastroesophageal adenocarcinoma. Cancer Biol Ther. 2011; 12: 9–46.

66. Biankin A V, Waddell N, Kassahn KS, Gingras M-C, Muthuswamy LB, Johns AL, Miller DK, Wilson PJ, Patch A-M, Wu J, Chang DK, Cowley MJ, Gardiner BB, et al. Pancreatic cancer genomes reveal aberrations in axon guidance pathway genes. Nature. 2012; 491: 399–405.

67. Ridge SA, Worwood M, Oscier D, Jacobs A, Padua RA. FMS mutations in myelodysplastic, leukemic, and normal subjects. Proc Natl Acad Sci U S A. 1990; 87: 1377–80.

68. Rademakers R, Baker M, Nicholson AM, Rutherford NJ, Finch N, Soto-Ortolaza A, Lash J, Wider C, Wojtas A, DeJesus-Hernandez M, Adamson J, Kouri N, Sundal C, et al. Mutations in the colony stimulating factor 1 receptor (CSF1R) gene cause hereditary diffuse leukoencephalopathy with spheroids. Nat Genet. 2011; 44: 200–5.

69. Reindl C, Bagrintseva K, Vempati S, Schnittger S, Ellwart JW, Wenig K, Hopfner K-P, Hiddemann W, Spiekermann K. Point mutations in the juxtamembrane domain of FLT3 define a new class of activating mutations in AML. Blood. 2006; 107: 3700–7.

70. Imielinski M, Berger AH, Hammerman PS, Hernandez B, Pugh TJ, Hodis E, Cho J, Suh J, Capelletti M, Sivachenko A, Sougnez C, Auclair D, Lawrence MS, et al. Mapping the hallmarks of lung adenocarcinoma with massively parallel sequencing. Cell. 2012; 150: 1107–20.

71. Smith CC, Wang Q, Chin C-S, Salerno S, Damon LE, Levis MJ, Perl AE, Travers KJ, Wang S, Hunt JP, Zarrinkar PP, Schadt EE, Kasarskis A, et al. Validation of ITD mutations in FLT3 as a therapeutic target in human acute myeloid leukaemia. Nature. 2012; 485: 260–3.

72. Yamamoto Y, Kiyoi H, Nakano Y, Suzuki R, Kodera Y, Miyawaki S, Asou N, Kuriyama K, Yagasaki F, Shimazaki C, Akiyama H, Saito K, Nishimura M, et al. Activating mutation of D835 within the activation loop of FLT3 in human hematologic malignancies. Blood. 2001; 97: 2434–9.

73. Taketani T, Taki T, Sugita K, Furuichi Y, Ishii E, Hanada R, Tsuchida M, Sugita K, Ida K, Hayashi Y. FLT3 mutations in the activation loop of tyrosine kinase domain are frequently found in infant ALL with MLL rearrangements and pediatric ALL with hyperdiploidy. Blood. 2004; 103: 1085–8.

74. Auewarakul CU, Sritana N, Limwongse C, Thongnoppakhun W, Yenchitsomanus P. Mutations of the FLT3 gene in adult acute myeloid leukemia: determination of incidence and identification of a novel mutation in a Thai population. Cancer Genet Cytogenet. 2005; 162: 127–34.

75. Antonescu CR, Sommer G, Sarran L, Tschernyavsky SJ, Riedel E, Woodruff JM, Robson M, Maki R, Brennan MF, Ladanyi M, DeMatteo RP, Besmer P. Association of KIT exon 9 mutations with nongastric primary site and aggressive behavior: KIT mutation analysis and clinical correlates of 120 gastrointestinal stromal tumors. Clin Cancer Res. 2003; 9: 3329–37.

76. Tetsu O, Phuchareon J, Chou A, Cox DP, Eisele DW, Jordan RCK. Mutations in the c-Kit gene disrupt mitogen-activated protein kinase signaling during tumor development in adenoid cystic carcinoma of the salivary glands. Neoplasia. 2010; 12: 708–17.

77. Tian Q, Frierson HFJ, Krystal GW, Moskaluk CA. Activating c-kit gene mutations in human germ cell tumors. Am J Pathol. 1999; 154: 1643–7. 78. Furitsu T, Tsujimura T, Tono T, Ikeda H, Kitayama H, Koshimizu U, Sugahara H, Butterfield JH, Ashman LK, Kanayama Y. Identification of mutations in the coding sequence of the proto-oncogene c-kit in a human mast cell leukemia cell line causing ligand-independent activation of c-kit product. J Clin Invest. 1993; 92: 1736–44.

79. Wardelmann E, Thomas N, Merkelbach-Bruse S, Pauls K, Speidel N, Buttner R, Bihl H, Leutner CC, Heinicke T, Hohenberger P. Acquired resistance to imatinib in gastrointestinal stromal tumours caused by multiple KIT mutations. Lancet Oncol. 2005; 6: 249–51.

80. Sakuma Y, Sakurai S, Oguni S, Satoh M, Hironaka M, Saito K. c-kit gene mutations in intracranial germinomas. Cancer Sci. 2004; 95: 716–20.

81. Kemmer K, Corless CL, Fletcher JA, McGreevey L, Haley A, Griffith D, Cummings OW, Wait C, Town A, Heinrich MC. KIT mutations are common in testicular seminomas. Am J Pathol. 2004; 164: 305–13.

82. Hongyo T, Li T, Syaifudin M, Baskar R, Ikeda H, Kanakura Y, Aozasa K, Nomura T. Specific c-kit mutations in sinonasal natural killer/T-cell lymphoma in China and Japan. Cancer Res. 2000; 60: 2345–7.

83. Longley BJJ, Metcalfe DD, Tharp M, Wang X, Tyrrell L, Lu SZ, Heitjan D, Ma Y. Activating and dominant inactivating c-KIT catalytic domain mutations in distinct clinical forms of human mastocytosis. Proc Natl Acad Sci U S A. 1999; 96: 1609–14.

84. Alentorn A, Marie Y, Carpentier C, Boisselier B, Giry M, Labussiere M, Mokhtari K, Hoang-Xuan K, Sanson M, Delattre J-Y, Idbaih A. Prevalence, clinico-pathological value, and co-occurrence of PDGFRA abnormalities in diffuse gliomas. Neuro Oncol. 2012; 14: 1393–403.

85. Hostein I, Debiec-Rychter M, Olschwang S, Bringuier P-P, Toffolati L, Gonzalez D, Forget S, Escande F, Morzuch L, Tamborini E, Faur N, Pilotti S, Dei Tos P, et al. A quality control program for mutation detection in KIT and PDGFRA in gastrointestinal stromal tumours. J Gastroenterol. 2011; 46: 586–94.

86. de Raedt T, Cools J, Debiec-Rychter M, Brems H, Mentens N, Sciot R, Himpens J, de Wever I, Schoffski P, Marynen P, Legius E. Intestinal neurofibromatosis is a subtype of familial GIST and results from a dominant activating mutation in PDGFRA. Gastroenterology. 2006; 131: 1907–12.

87. Heinrich MC, Corless CL, Duensing A, McGreevey L, Chen C-J, Joseph N, Singer S, Griffith DJ, Haley A, Town A, Demetri GD, Fletcher CDM, Fletcher JA. PDGFRA activating mutations in gastrointestinal stromal tumors. Science. 2003; 299: 708–10.

88. Dai J, Kong Y, Si L, Chi Z, Cui C, Sheng X, Mao L, Li S, Lian B, Yang R, Liu S, Xu X, Guo J. Large-scale analysis of PDGFRA mutations in melanomas and evaluation of their sensitivity to tyrosine kinase inhibitors imatinib and crenolanib. Clin Cancer Res. 2013; 19: 6935–42.

89. Cools J, DeAngelo DJ, Gotlib J, Stover EH, Legare RD, Cortes J, Kutok J, Clark J, Galinsky I, Griffin JD, Cross NCP, Tefferi A, Malone J, et al. A tyrosine kinase created by fusion of the PDGFRA and FIP1L1 genes as a therapeutic target of imatinib in idiopathic hypereosinophilic syndrome. N Engl J Med. 2003; 348: 1201–14.

90. Chompret A, Kannengiesser C, Barrois M, Terrier P, Dahan P, Tursz T, Lenoir GM, Bressac-De Paillerets B. PDGFRA germline mutation in a family with multiple cases of gastrointestinal stromal tumor. Gastroenterology. 2004; 126: 318–21.

91. Elling C, Erben P, Walz C, Frickenhaus M, Schemionek M, Stehling M, Serve H, Cross NCP, Hochhaus A, Hofmann W-K, Berdel WE, Muller-Tidow C, Reiter A, et al. Novel imatinib-sensitive PDGFRA-activating point mutations in hypereosinophilic syndrome induce growth factor independence and leukemia-like disease. Blood. 2011; 117: 2935–43.

92. Cheung YH, Gayden T, Campeau PM, LeDuc CA, Russo D, Nguyen V-H, Guo J, Qi M, Guan Y, Albrecht S, Moroz B, Eldin KW, Lu JT, et al. A recurrent PDGFRB mutation causes familial infantile myofibromatosis. Am J Hum Genet. 2013; 92: 996–1000.

93. Wagle N, Van Allen EM, Treacy DJ, Frederick DT, Cooper ZA, Taylor-Weiner A, Rosenberg M, Goetz EM, Sullivan RJ, Farlow DN, Friedrich DC, Anderka K, Perrin D, et al. MAP kinase pathway alterations in BRAF-mutant melanoma patients with acquired resistance to combined RAF/MEK inhibition. Cancer Discov. 2014; 4: 61–8.

94. Nicolas G, Pottier C, Maltete D, Coutant S, Rovelet-Lecrux A, Legallic S, Rousseau S, Vaschalde Y, Guyant-Marechal L, Augustin J, Martinaud O, Defebvre L, Krystkowiak P, et al. Mutation of the PDGFRB gene as a cause of idiopathic basal ganglia calcification. Neurology. 2013; 80: 181–7.

95. Chiara F, Bishayee S, Heldin C-H, Demoulin J-B. Autoinhibition of the platelet-derived growth factor beta-receptor tyrosine kinase by its C-terminal tail. J Biol Chem. 2004; 279: 19732–8.

96. Decker RA, Peacock ML, Watson P. Hirschsprung disease in MEN 2A: increased spectrum of RET exon 10 genotypes and strong genotype-phenotype correlation. Hum Mol Genet. 1998; 7: 129–34.

97. Caron P, Attie T, David D, Amiel J, Brousset F, Roger P, Munnich A, Lyonnet S. C618R mutation in exon 10 of the RET proto-oncogene in a kindred with multiple endocrine neoplasia type 2A and Hirschsprung’s disease. J Clin Endocrinol Metab. 1996; 81: 2731–3.

98. Lore F, Talidis F, Di Cairano G, Renieri A. Multiple endocrine neoplasia type 2 syndromes may be associated with renal malformations. J Intern Med. 2001; 250: 37–42.

99. Crona J, Nordling M, Maharjan R, Granberg D, Stalberg P, Hellman P, Bjorklund P. Integrative genetic characterization and phenotype correlations in pheochromocytoma and paraganglioma tumours. PLoS One. 2014; 9: e86756.

100. Mulligan LM, Eng C, Healey CS, Clayton D, Kwok JB, Gardner E, Ponder MA, Frilling A, Jackson CE, Lehnert H. Specific mutations of the RET proto-oncogene are related to disease phenotype in MEN 2A and FMTC. Nat Genet. 1994; 6: 70–4.

101. Neumann HPH, Bausch B, McWhinney SR, Bender BU, Gimm O, Franke G, Schipper J, Klisch J, Altehoefer C, Zerres K, Januszewicz A, Eng C, Smith WM, et al. Germ-line mutations in nonsyndromic pheochromocytoma. N Engl J Med. 2002; 346: 1459–66.

102. Yang Y, Houle A-M, Letendre J, Richter A. RET Gly691Ser mutation is associated with primary vesicoureteral reflux in the French-Canadian population from Quebec. Hum Mutat. 2008; 29: 695–702.

103. Chatterjee R, Ramos E, Hoffman M, VanWinkle J, Martin DR, Davis TK, Hoshi M, Hmiel SP, Beck A, Hruska K, Coplen D, Liapis H, Mitra R, et al. Traditional and targeted exome sequencing reveals common, rare and novel functional deleterious variants in RET-signaling complex in a cohort of living US patients with urinary tract malformations. Hum Genet. 2012; 131: 1725–38.

104. Lesueur F, Cebrian A, Cranston A, Leyland J, Faid TM, Clements MR, Robledo M, Whittaker J, Ponder BAJ. Germline homozygous mutations at codon 804 in the RET protooncogene in medullary thyroid carcinoma/multiple endocrine neoplasia type 2A patients. J Clin Endocrinol Metab. 2005; 90: 3454–7.

105. Jimenez C, Dang GT, Schultz PN, El-Naggar A, Shapiro S, Barnes EA, Evans DB, Vassilopoulou-Sellin R, Gagel RF, Cote GJ, Hoff AO. A novel point mutation of the RET protooncogene involving the second intracellular tyrosine kinase domain in a family with medullary thyroid carcinoma. J Clin Endocrinol Metab. 2004; 89: 3521–6.

106. Carlson KM, Bracamontes J, Jackson CE, Clark R, Lacroix A, Wells SAJ, Goodfellow PJ. Parent-of-origin effects in multiple endocrine neoplasia type 2B. Am J Hum Genet. 1994; 55: 1076–82.

107. Comprehensive molecular characterization of human colon and rectal cancer. Nature. 2012; 487: 330–7.

108. Leich E, Weissbach S, Klein H-U, Grieb T, Pischimarov J, Stuhmer T, Chatterjee M, Steinbrunn T, Langer C, Eilers M, Knop S, Einsele H, Bargou R, et al. Multiple myeloma is affected by multiple and heterogeneous somatic mutations in adhesion- and receptor tyrosine kinase signaling molecules. Blood Cancer J. 2013; 3: e102.

109. Stransky N, Egloff AM, Tward AD, Kostic AD, Cibulskis K, Sivachenko A, Kryukov G V, Lawrence MS, Sougnez C, McKenna A, Shefler E, Ramos AH, Stojanov P, et al. The mutational landscape of head and neck squamous cell carcinoma. Science. 2011; 333: 1157–60.

110. Peifer M, Fernandez-Cuesta L, Sos ML, George J, Seidel D, Kasper LH, Plenker D, Leenders F, Sun R, Zander T, Menon R, Koker M, Dahmen I, et al. Integrative genome analyses identify key somatic driver mutations of small-cell lung cancer. Nat Genet. 2012; 44: 1104–10.

111. Berger MF, Hodis E, Heffernan TP, Deribe YL, Lawrence MS, Protopopov A, Ivanova E, Watson IR, Nickerson E, Ghosh P, Zhang H, Zeid R, Ren X, et al. Melanoma genome sequencing reveals frequent PREX2 mutations. Nature. 2012; 485: 502–6.

112. Vikkula M, Boon LM, Carraway KL, Calvert JT, Diamonti AJ, Goumnerov B, Pasyk KA, Marchuk DA, Warman ML, Cantley LC, Mulliken JB, Olsen BR. Vascular dysmorphogenesis caused by an activating mutation in the receptor tyrosine kinase TIE2. Cell. 1996; 87: 1181–90.

113. Wouters V, Limaye N, Uebelhoer M, Irrthum A, Boon LM, Mulliken JB, Enjolras O, Baselga E, Berg J, Dompmartin A, Ivarsson SA, Kangesu L, Lacassie Y, et al. Hereditary cutaneomucosal venous malformations are caused by TIE2 mutations with widely variable hyper-phosphorylating effects. Eur J Hum Genet. 2010; 18: 414–20.

114. Limaye N, Wouters V, Uebelhoer M, Tuominen M, Wirkkala R, Mulliken JB, Eklund L, Boon LM, Vikkula M. Somatic mutations in angiopoietin receptor gene TEK cause solitary and multiple sporadic venous malformations. Nat Genet. 2009; 41: 118–24.

115. Welch JS, Ley TJ, Link DC, Miller CA, Larson DE, Koboldt DC, Wartman LD, Lamprecht TL, Liu F, Xia J, Kandoth C, Fulton RS, McLellan MD, et al. The origin and evolution of mutations in acute myeloid leukemia. Cell. 2012; 150: 264–78.

116. Kim T-M, Jung S-H, Kim MS, Baek I-P, Park S-W, Lee SH, Lee HH, Kim SS, Chung Y-J, Lee SH. The mutational burdens and evolutionary ages of early gastric cancers are comparable to those of advanced gastric cancers. J Pathol. 2014; 234: 365–74.

117. Witkiewicz AK, McMillan EA, Balaji U, Baek G, Lin W-C, Mansour J, Mollaee M, Wagner K-U, Koduru P, Yopp A, Choti MA, Yeo CJ, McCue P, et al. Whole-exome sequencing of pancreatic cancer defines genetic diversity and therapeutic targets. Nat Commun. 2015; 6: 6744.

118. Rudin CM, Durinck S, Stawiski EW, Poirier JT, Modrusan Z, Shames DS, Bergbower EA, Guan Y, Shin J, Guillory J, Rivers CS, Foo CK, Bhatt D, et al. Comprehensive genomic analysis identifies SOX2 as a frequently amplified gene in small-cell lung cancer. Nat Genet. 2012; 44: 1111–6.

119. Gray J, Yeo G, Hung C, Keogh J, Clayton P, Banerjee K, McAulay A, O’Rahilly S, Farooqi IS. Functional characterization of human NTRK2 mutations identified in patients with severe early-onset obesity. Int J Obes. 2007; 31: 359–64.

120. Wood LD, Calhoun ES, Silliman N, Ptak J, Szabo S, Powell SM, Riggins GJ, Wang T-L, Yan H, Gazdar A, Kern SE, Pennacchio L, Kinzler KW, et al. Somatic mutations of GUCY2F, EPHA3, and NTRK3 in human cancers. Hum Mutat. 2006; 27: 1060–1.

121. Bardelli A, Parsons DW, Silliman N, Ptak J, Szabo S, Saha S, Markowitz S, Willson JK V, Parmigiani G, Kinzler KW, Vogelstein B, Velculescu VE. Mutational analysis of the tyrosine kinome in colorectal cancers. Science. 2003; 300: 949.

122. Wang K, Yuen ST, Xu J, Lee SP, Yan HHN, Shi ST, Siu HC, Deng S, Chu KM, Law S, Chan KH, Chan ASY, Tsui WY, et al. Whole-genome sequencing and comprehensive molecular profiling identify new driver mutations in gastric cancer. Nat Genet. 2014; 46: 573–82.

123. Frattini V, Trifonov V, Chan JM, Castano A, Lia M, Abate F, Keir ST, Ji AX, Zoppoli P, Niola F, Danussi C, Dolgalev I, Porrati P, et al. The integrated landscape of driver genomic alterations in glioblastoma. Nat Genet. 2013; 45: 1141–9.

124. Hunter C, Smith R, Cahill DP, Stephens P, Stevens C, Teague J, Greenman C, Edkins S, Bignell G, Davies H, O’Meara S, Parker A, Avis T, et al. A hypermutation phenotype and somatic MSH6 mutations in recurrent human malignant gliomas after alkylator chemotherapy. Cancer Res. 2006; 66: 3987–91.

125. Krauthammer M, Kong Y, Ha BH, Evans P, Bacchiocchi A, McCusker JP, Cheng E, Davis MJ, Goh G, Choi M, Ariyan S, Narayan D, Dutton-Regester K, et al. Exome sequencing identifies recurrent somatic RAC1 mutations in melanoma. Nat Genet. 2012; 44: 1006–14.

126. Jinnin M, Medici D, Park L, Limaye N, Liu Y, Boscolo E, Bischoff J, Vikkula M, Boye E, Olsen BR. Suppressed NFAT-dependent VEGFR1 expression and constitutive VEGFR2 signaling in infantile hemangioma. Nat Med. 2008; 14: 1236–46.

127. Parsons DW, Jones S, Zhang X, Lin JC-H, Leary RJ, Angenendt P, Mankoo P, Carter H, Siu I-M, Gallia GL, Olivi A, McLendon R, Rasheed BA, et al. An integrated genomic analysis of human glioblastoma multiforme. Science. 2008; 321: 1807–12.

128. Walter JW, North PE, Waner M, Mizeracki A, Blei F, Walker JWT, Reinisch JF, Marchuk DA. Somatic mutation of vascular endothelial growth factor receptors in juvenile hemangioma. Genes Chromosomes Cancer. 2002; 33: 295–303.

129. Nikolaev SI, Rimoldi D, Iseli C, Valsesia A, Robyr D, Gehrig C, Harshman K, Guipponi M, Bukach O, Zoete V, Michielin O, Muehlethaler K, Speiser D, et al. Exome sequencing identifies recurrent somatic MAP2K1 and MAP2K2 mutations in melanoma. Nat Genet. 2011; 44: 133–9.

130. Evans AL, Bell R, Brice G, Comeglio P, Lipede C, Jeffery S, Mortimer P, Sarfarazi M, Child AH. Identification of eight novel VEGFR-3 mutations in families with primary congenital lymphoedema. J Med Genet. 2003; 40: 697 LP-703.

131. Irrthum A, Karkkainen MJ, Devriendt K, Alitalo K, Vikkula M. Congenital Hereditary Lymphedema Caused by a Mutation That Inactivates VEGFR3 Tyrosine Kinase. Am J Hum Genet. 2000; 67: 295–301.

132. Spiegel R, Ghalamkarpour A, Daniel-Spiegel E, Vikkula M, Shalev SA. Wide clinical spectrum in a family with hereditary lymphedema type I due to a novel missense mutation in VEGFR3. J Hum Genet. 2006; 51: 846–50.

133. Stephens PJ, Tarpey PS, Davies H, Van Loo P, Greenman C, Wedge DC, Nik-Zainal S, Martin S, Varela I, Bignell GR, Yates LR, Papaemmanuil E, Beare D, et al. The landscape of cancer genes and mutational processes in breast cancer. Nature. 2012; 486: 400–4.
