# Supplementary material for: Inhibitor repurposing reveals ALK, LTK, FGFR, RET and TRK kinases as the targets of AZD1480
Source: Oncotarget. 2017 Nov 27;8(65):109319–31. doi: 10.18632/oncotarget.22674 (PMC5752523; doi:10.18632/oncotarget.22674)
Supplement: Supplementary file 4 [file oncotarget-08-109319-s004.doc]

Supplementary Table 3: Kinase-dead RTK mutants generated in the study

| *RTK* | *Mutation* | *References* |
| --- | --- | --- |
| ALK | I1250T | [1] |
| LTK | K544M | [2] |
| AXL | K567M | [2,3] |
| DDR1 | K655M | [2,4] |
| DDR2 | K608M | [2,5] |
| ERBB2 | K753M | [2,6] |
| ERBB4 | K751M | [2,7] |
| FGFR2 | A649T | [8] |
| FGFR3 | K508M | [9] |
| FGFR4 | K503M | [2,10] |
| INSR | A1162E | [11] |
| INSRR | K1013M | [2] |
| MET | K1110A | [12] |
| RON | K1114M | [2,13] |
| CSF1R | K616M | [2] |
| FLT3 | K644M | [2] |
| KIT | K623M | [2,14] |
| PDGFRA | K627M | [2] |
| PDGFRB | K634A | [15] |
| RET | K758M | [2,16] |
| ROS1 | K1980M | [2] |
| TEK | K855M | [2] |
| TIE | K870M | [2] |
| TRKA | G577R | [17] |
| TRKB | Y722C | [18] |
| TRKC | K572M | [2] |
| VEGFR1 | K861M | [2] |
| VEGFR2 | K868M | [2,19] |
| VEGFR3 | K879M | [2] |

References

1. Schönherr C, Ruuth K, Eriksson T, Yamazaki Y, Ottmann C, Combaret V, Vigny M, Kamaraj S, Palmer RH, Hallberg B. The Neuroblastoma ALK(I1250T) Mutation Is a Kinase-Dead RTK In Vitro and In Vivo. Transl Oncol. 2011; 4: 258–IN6.

2. Varjosalo M, Björklund M, Cheng F, Syvänen H, Kivioja T, Kilpinen S, Sun Z, Kallioniemi O, Stunnenberg HG, He WW, Ojala P, Taipale J. Application of Active and Kinase-Deficient Kinome Collection for Identification of Kinases Regulating Hedgehog Signaling. Cell. 2008; 133: 537–48.

3. Braunger J, Schleithoff L, Schulz a S, Kessler H, Lammers R, Ullrich A, Bartram CR, Janssen JW. Intracellular signaling of the Ufo/Axl receptor tyrosine kinase is mediated mainly by a multi-substrate docking-site. Oncogene. 1997; 14: 2619–31.

4. Rix U, Hantschel O, Dürnberger G, Remsing Rix LL, Planyavsky M, Fernbach N V., Kaupe I, Bennett KL, Valent P, Colinge J, Köcher T, Superti-Furga G. Chemical proteomic profiles of the BCR-ABL inhibitors imatinib, nilotinib, and dasatinib reveal novel kinase and nonkinase targets. Blood. 2007; 110: 4055–63.

5. Iwai LK, Payne LS, Luczynski MT, Chang F, Xu H, Clinton RW, Paul A, Esposito EA, Gridley S, Leitinger B, Naegle KM, Huang PH. Phosphoproteomics of collagen receptor networks reveals SHP-2 phosphorylation downstream of wild-type DDR2 and its lung cancer mutants. Biochem J. 2013; 454: 501–13.

6. Tan M, Li P, Klos KS, Lu J, Lan KH, Nagata Y, Fang D, Jing T, Yu D. ErbB2 promotes Src synthesis and stability: Novel mechanisms of Src activation that confer breast cancer metastasis. Cancer Res. 2005; 65: 1858–67.

7. Yang XL, Huang YZ, Xiong WC, Mei L. Neuregulin-induced expression of the acetylcholine receptor requires endocytosis of ErbB receptors. Mol Cell Neurosci. 2005; 28: 335–46.

8. Gallo LH, Nelson KN, Meyer AN, Donoghue DJ. Functions of Fibroblast Growth Factor Receptors in cancer defined by novel translocations and mutations. Cytokine Growth Factor Rev. 2015; 26: 425–49.

9. Raffioni S, Zhu YZ, Brashaw RA, Thompson LM. Effect of transmembrane and kinase domain mutations on fibroblast growth factor receptor 3 chimera signaling in PC12 cells. A model for the control of receptor tyrosine kinase activation. J Biol Chem. 1998; 273: 35250–9.

10. Fawdar S, Trotter EW, Li Y, Stephenson NL, Hanke F, Marusiak A a, Edwards ZC, Ientile S, Waszkowycz B, Miller CJ, Brognard J. Targeted genetic dependency screen facilitates identification of actionable mutations in FGFR4, MAP3K9, and PAK5 in lung cancer. Proc Natl Acad Sci U S A. 2013; 110: 12426–31.

11. Ardon O, Procter M, Tvrdik T, Longo N, Mao R. Sequencing analysis of insulin receptor defects and detection of two novel mutations in INSR gene. Mol Genet Metab Reports. 2014; 1: 71–84.

12. Zhu H, Naujokas MA, Fixman ED, Torossian K, Park M. Tyrosine 1356 in the carboxyl-terminal tail of the HGF/SF receptor is essential for the transduction of signals for cell motility and morphogenesis. J Biol Chem . 1994; 269: 29943–8.

13. Danilkovitch-Miagkova A, Angeloni D, Skeel A, Donley S, Lerman M, Leonard EJ. Integrin-mediated RON Growth Factor Receptor Phosphorylation Requires Tyrosine Kinase Activity of Both the Receptor and c-Src. J Biol Chem . 2000; 275: 14783–6.

14. Mancini A, Koch A, Stefan M, Niemann H, Tamura T. The direct association of the multiple PDZ domain containing proteins (MUPP-1) with the human c-Kit C-terminus is regulated by tyrosine kinase activity. FEBS Lett. 2000; 482: 54–8.

15. Emaduddin M, Ekman S, Ronnstrand L, Heldin CH. Functional co-operation between the subunits in heterodimeric platelet-derived growth factor receptor complexes. Biochem J. 1999; 341: 523–8.

16. Liu X, Vega QC, Decker RA, Pandey A, Worby CA, Dixon JE. Oncogenic RET receptors display different autophosphorylation sites and substrate binding specificities. J Biol Chem. 1996; 271: 5309–12.

17. Miranda C, Mazzoni M, Sensi M, Pierotti MA, Greco A. Functional characterization of NTRK1 mutations identified in melanoma. Genes Chromosom Cancer. 2014; 53: 875–80.

18. Gray J, Yeo G, Hung C, Keogh J, Clayton P, Banerjee K, McAulay A, O’Rahilly S, Farooqi IS. Functional characterization of human NTRK2 mutations identified in patients with severe early-onset obesity. Int J Obes. 2007; 31: 359–64.

19. Takahashi T, Yamaguchi S, Chida K, Shibuya M. A single autophosphorylation site on KDR/Flk‐1 is essential for VEGF‐A‐dependent activation of PLC‐γ and DNA synthesis in vascular endothelial cells. EMBO J. 2001; 20: 2768 LP-2778.
